# Progress of Nanomaterials in Photodynamic Therapy Against Tumor

**DOI:** 10.3389/fbioe.2022.920162

**Published:** 2022-05-31

**Authors:** Lei Chen, Jiahui Huang, Xiaotong Li, Miaoting Huang, Shaoting Zeng, Jiayi Zheng, Shuyi Peng, Shiying Li

**Affiliations:** ^1^ Department of Anesthesiology, The First Affiliated Hospital of Guangzhou Medical University, Guangzhou, China; ^2^ Department of Anesthesiology, Huizhou Central People’s Hospital, Huizhou, China; ^3^ Guangzhou Medical University, Guangzhou, China; ^4^ Key Laboratory of Molecular Target and Clinical Pharmacology and The State Key Laboratory of Respiratory Disease, School of Pharmaceutical Sciences and The Fifth Affiliated Hospital, Guangzhou Medical University, Guangzhou, China

**Keywords:** photodynamic therapy, tumor microenvironment, nanomaterials, tumor-targeting, photosensitizers

## Abstract

Photodynamic therapy (PDT) is an advanced therapeutic strategy with light-triggered, minimally invasive, high spatiotemporal selective and low systemic toxicity properties, which has been widely used in the clinical treatment of many solid tumors in recent years. Any strategies that improve the three elements of PDT (light, oxygen, and photosensitizers) can improve the efficacy of PDT. However, traditional PDT is confronted some challenges of poor solubility of photosensitizers and tumor suppressive microenvironment. To overcome the related obstacles of PDT, various strategies have been investigated in terms of improving photosensitizers (PSs) delivery, penetration of excitation light sources, and hypoxic tumor microenvironment. In addition, compared with a single treatment mode, the synergistic treatment of multiple treatment modalities such as photothermal therapy, chemotherapy, and radiation therapy can improve the efficacy of PDT. This review summarizes recent advances in nanomaterials, including metal nanoparticles, liposomes, hydrogels and polymers, to enhance the efficiency of PDT against malignant tumor.

## 1 Introduction

PDT mainly relies on PSs to generate ^1^O_2_ from O_2_ under the induction of specific wavelengths of light, causing oxidative damage to tumor cells and killing them, and even triggering immunogenic cell death (ICD). However, the insufficient supply of key factors such as PSs, light, and O_2_ in tumor tissue greatly reduces the therapeutic effect of PDT (Lee et al., 2022). Nanoparticles (NPs) as drug carriers have received extensive attention in the field of tumor therapy. They can achieve high-efficiency delivery of PSs to tumor tissues through physicochemically optimized passive targeting, ligand-modified active targeting, and stimulus-responsive release (Zhao et al., 2021). Attempts have been made to overcome the unfavorable tumor microenvironment through photocatalytic oxygen production, Fenton reaction, and combination with other chemical drugs (Wan et al., 2021). In this review, we first describe the composition and tumor-promoting mechanisms of the tumor microenvironment, and then introduce metal NPs, nanoliposomes, mesoporous silica NPs, dendrimers, hydrogels, polymer micelles and these creative methods to solve the problems faced by tumor PDT. In particular, in this review, we focus on recent advances in diverse metal NPs including metal-organic frameworks (MOF), which provide a promising approach for the design of integrative therapeutics in clinical treatments.

## 2 Tumor Microenvironment

**FIGURE 1:**
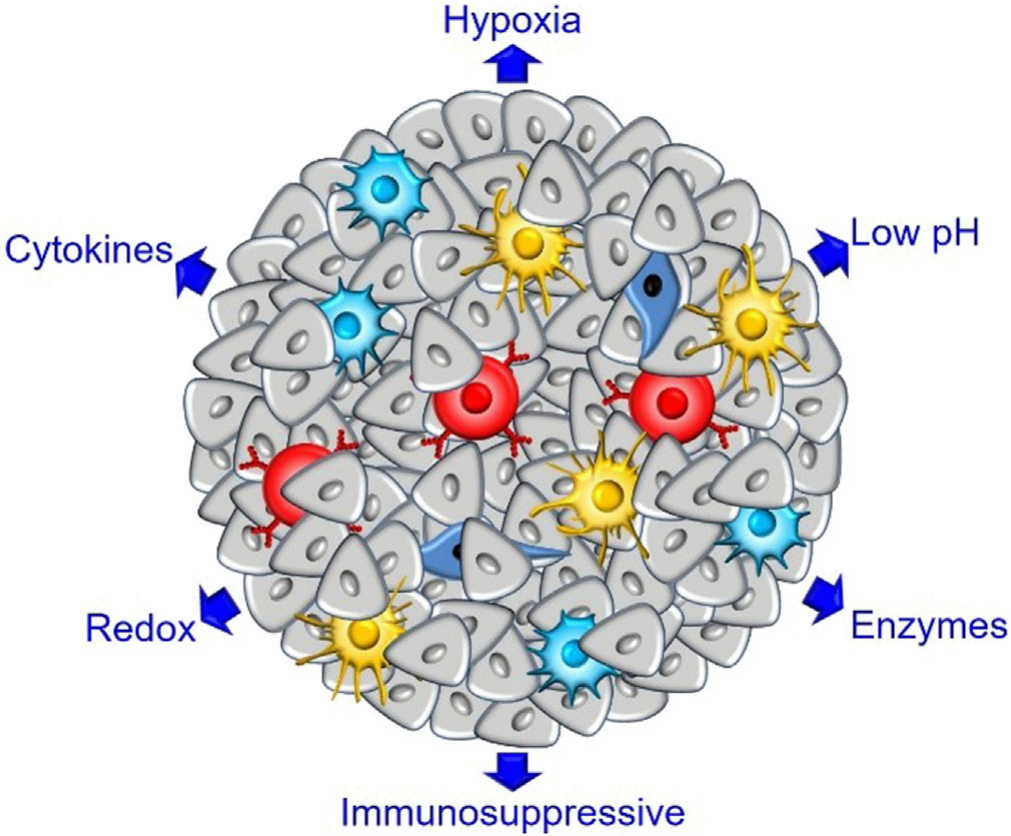
Schematic representation of the tumor microenvironment in solid tumors.

TME refers to the non-cancerous cells and components presented in the tumor, including blood vessels, extracellular matrix (ECM), fibroblasts, the surrounding immune cells, molecules produced and released by them (Figure 1) (Chang et al., 2021). Hypoxic and acidic microenvironment, caused by tumor vascular tissue distribution disorder and structural abnormality, is the most important supporting component in TME and immunosuppressive microenvironment and has offered a favorable niche for tumor growth, proliferation and invasion (Yuan X. et al., 2022). The immune cells, including granulocytes, lymphocytes, and macrophages, are involved in various immune responses and activities orchestrated by the tumor to promote tumor survival. Among them, the macrophages abundantly infiltrating TME are called tumor-associated macrophages (tumor-associated macrophages, TAMs), which are the most prominent immune cell type in the TME. According to the difference of phenotype and function, activated macrophages can be divided into M1 and M2, and their polarization direction is regulated by microenvironment (Wang et al., 2021a). For example, tissue microenvironment, external factors and inflammatory response factors can activate macrophages in different forms (Cenowicz et al., 2021). The macrophages that promote tumor growth are M2 phenotype and have the function of repairing injury and inhibiting inflammatory response in normal tissues. The macrophages that inhibit tumor growth are M1 phenotype, which can induce inflammatory response and activate immune response to kill tumor cells (Chen J. et al., 2021). In the process of tumorigenesis, TAMs can often stimulate angiogenesis, promote tumor cell migration and invasion, and mediate tumor immune escape (De Lerma et al., 2021). In the site of tumor metastasis, TAMs promote tumor cell exosmosis, survival and follow-up activity. At the same time, TAMs affect the clinical therapeutic effect of tumors by enhancing the genetic instability of tumor cells, nourishing tumor stem cells, and promoting infiltration and metastasis, and they are the key driving force for tumor growth. The degree of macrophage infiltration in tumor tissues is related to the poor prognosis of patients, and the number of TAMs is negatively related to the survival time of patients (Munir et al., 2021). In addition, an increase in neutrophils in blood also accounts for a sign of poor prognosis for cancer. Imitating the naming method used to define macrophages (M1-like and M2-like/TAM), tumor-associated neutrophils (TANs) can obtain at least two different phenotypes: N1 neutrophils, endowed with anti-tumor activities, and N2 neutrophils endowed with immunosuppressive and pro-angiogenic properties to support tumor progression (Li et al., 2020). The tumor-promoting activity of TAN can cover a variety of mechanisms (Taucher et al., 2021). TAN can’t only promote tumor angiogenesis by secreting matrix metalloproteinase-9 (MMP-9) and vascular endothelial growth factor (VEGF) from the extracellular matrix (ECM), but also inhibit CD8T cells and produce immunosuppressive environment by secreting arginase 1 (Xiao et al., 2021).

Cancer cells harbor a different metabolic profile with respect to healthy cells (Chou and Yang, 2021). Cancer cells can maintain a high rate of glycolysis even in the presence of O_2_, consume large amounts of glucose and significant areas in tumors exhibit lactic acidosis. This phenomenon known as “aerobic glycolysis” or the “Warburg effect” (Yuan et al., 2021). The tumor-promoting mechanisms of TME include: 1) HIF-1α nuclear transport pathway: Hypoxia inducible factor-1 (HIF-1) is a key transcriptional activator responsible for regulating target genes that contribute to survival and growth of cells in hypoxia condition (You et al., 2021). It consists of two subunits (HIF-1β and HIF-1α) and HIF-1α is sensitive to hypoxia. Under hypoxic conditions, HIF-1 becomes a stable and transcriptionally active dimer and then induces transcriptional and post-transcriptional regulation of target genes. In addition, overexpression of HIF-1α also promotes the transcription and activation of angiogenic factors, such as angiogenin, platelet-derived growth factors, plasminogen activator inhibitors and VEGF (Sun et al., 2022). 2) STAT3 phosphorylation pathway: Acidic TME inhibits T cell activation and cytotoxicity by inducing STAT3 phosphorylation (Liang et al., 2021). 3) mTOR signaling pathway: The persistent stress of hypoxic TME leads to excessive activation of mTOR signaling in NK cells, mitochondrial fission, and impaired metabolism, ultimately leading to NK cell depletion and reduced anti-tumor ability (Liang et al., 2021). 4) VEGF pathway and nuclear transcription factor (NF-κB) pathway: Enriching cytokines such as VEGF and eosinophil chemokine (Eotaxin) in tumor tissue, inducing macrophage polarization into M2 type. M2 TAMs further produce factors such as CC motif chemokine 2 (CCL2), CC motif chemokine 5 (CCL5) or macrophage colony stimulating factor 1 (CSF-1) to participate in immunosuppression (Huang et al., 2021). 5) Promote the production of factors such as hyaluronic acid (HA) and VEGF, thereby maintaining tumor growth and migration (Niu et al., 2021).

## 3 Photodynamic Therapy

Taking advantage of the fact that PSs can’t generate ROS in the dark environment, and is not toxic to cells and tissues. By finely controlling the illumination area, the PDT process can be confined within the tumor tissue, achieving highly selective killing of tumor cells and reducing the side effects of normal cell death (Lee et al., 2020). According to the different types and production methods of ROS, PDT can be divided into two mechanisms, type I and type II (Algorri et al., 2021). Type I reactions can directly react with biomolecules to produce radicals by transferring protons or electrons. In Type II reactions, the excited PS transfers energy to oxygen molecules to produce singlet oxygen (^1^O_2_). Both oxygen-containing free radicals and ^1^O_2_ have extremely high reactivity, which can damage a variety of biomolecules and kill tumor cells. Type II PDT requires PSs to generate ROS. However, PSs are often excited by visible light, which limits the efficacy of PDT for deep tumors. Furthermore, due to the short intracellular half-life of ROS (≈10–320 ns), the light penetration depth and the distribution of PSs in tumor tissue limit the efficacy of PDT (Lee et al., 2022). Nanomaterials provide a powerful tool to overcome many drawbacks of PSs in cancer PDT, such as hydrophobicity, short blood circulation time after intravenous injection, which lead to insufficient accumulation, retention and internalization in tumor tissues (Silva et al., 2021). Furthermore, some multifunctional nanomaterials can increase the levels of O_2_ and ROS in tissues by mediating photocatalytic oxygen production and Fenton reaction. In addition, upconverting NPs can enhance light delivery in tumor tissues by converting more penetrating NIR to visible light or preparing them as persistent luminescent NPs (Liu B. et al., 2021). In addition to enhancing PSs in tumor tissues through physicochemically optimized passive targeting, ligand-modified active targeting, and stimulus-responsive release. Nanomaterials can also be combined with chemotherapy, gene therapy, immunotherapy, photothermal therapy, hyperthermia/magnetothermal therapy, radiotherapy, sonodynamic therapy to overcome the limitations of PDT (Yang G. et al., 2021; Zhang P. et al., 2022). This study focuses on the commonly used PSs nanomodification techniques and the application of different types of novel nanomaterials for cancer treatment and diagnosis, such as nanoparticles, liposomes, hydrogels, polymers, etc.

## 4 Strategies for Nanoparticle-Based Photocontrolled Delivery

### 4.1 Metal Nanoparticles

Among the NPs, metal NPs have the advantages of high biocompatibility and stability, adjustable size, good optical properties, easy surface functionalization, and a long activity period (Desai et al., 2021). They can be used as PSs, delivery carriers and up-conversion tools to improve the delivery of chemotherapy, radionuclides and antibody drugs to tumor cells.

#### 4.1.1 Metal Based Nanoparticles

##### 4.1.1.1 Au Based Nanoparticles

Au NPs have surface plasmon resonance (SPR), chemical inertness and excellent biocompatibility, which are mainly used as passivation agents, drug delivery agents, imaging agents, and photothermal agents, which have different characteristic shapes, such as particles, rod-shaped, cluster-shaped, shell-shaped, spike-shaped and star, etc., (Younis et al., 2021). In PDT, gold nanoparticles can be used alone or as part of a multifunctional nanomaterial hybrid system for PSs delivery.

**FIGURE 2:**
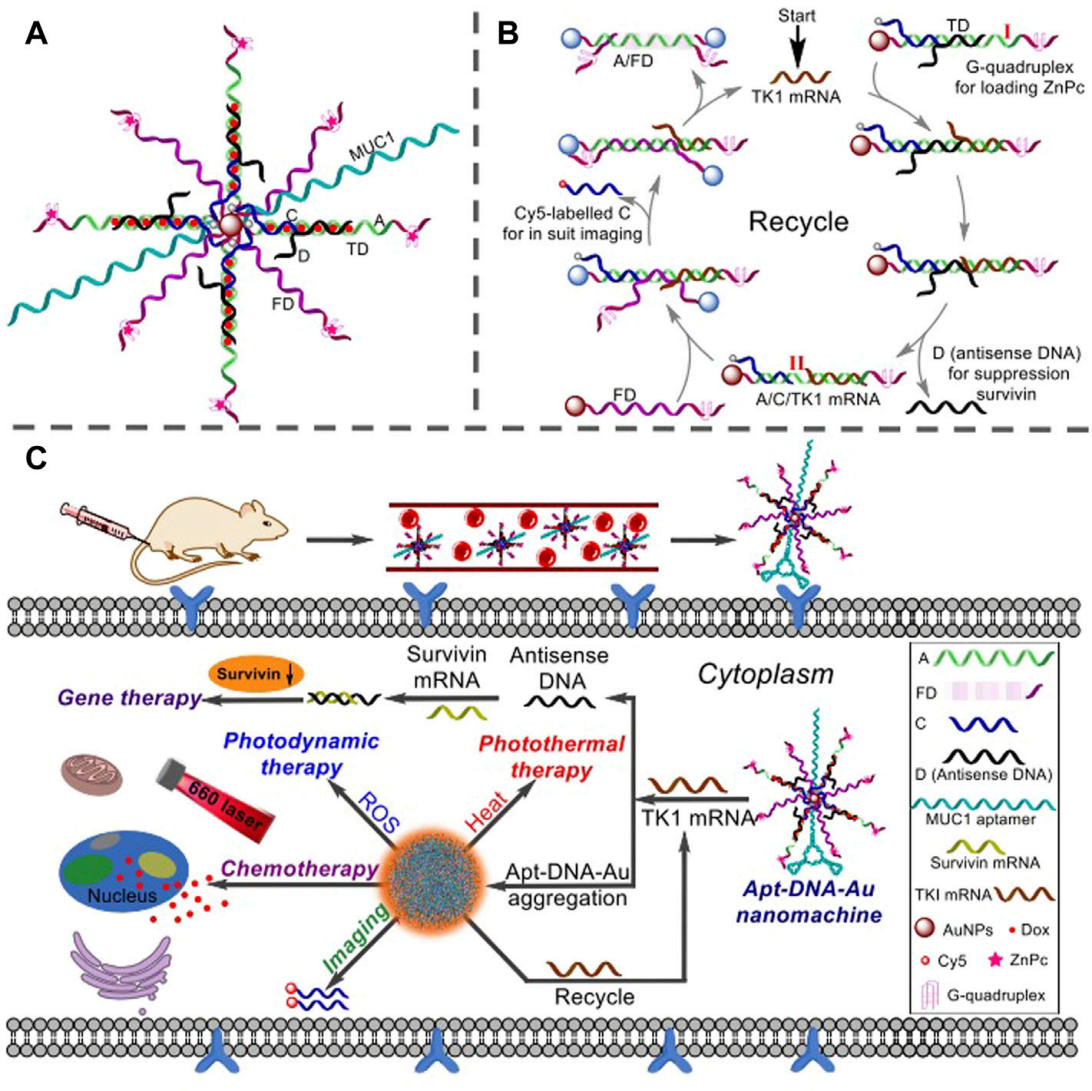
Multifunctional Apt-DNA-Au nanomachines for *in situ* imaging and targeted multimodal synergistic cancer therapy. **(A)** Schematic diagram of the structure of the Apt-DNA-Au nanomachine. MUC1 segment is an aptamer that specifically recognizes MCF-7 cells; A segment and Fd segment contain G-quadruplex structure for loading photosensitizer (ZnPc); C segment is labeled with Cy5 for *in situ* fluorescence imaging; D segment is incorporated into antisense DNA that inhibits Survivin expression for inhibiting tumor cell proliferation; TD segment is an A/C/D mixture loaded with Dox. **(B)** Working principle of the Apt-DNA-Au nanomachine. **(C)** Apt-DNA-Au nanomachines specifically recognize and internalize to target cancer cells, and monitor tumor therapeutic effects in real time through *in situ* fluorescence imaging and multimodal anticancer therapy (combined with chemotherapy, gene therapy, PDT, and PTT). Reproduced with permission from (Yu et al., 2021).

DNA is considered to be one of the best components for building nanomaterials due to its excellent sequence specificity and programmable supramolecular self-assembly. Among them, functional modification of nanomaterials can significantly improve the cellular stability of DNA nanomaterials. Based on this, Yu et al. utilized doxorubicin (Dox), antisense DNA (target Survivin mRNA) that could inhibit Survivin expression, photosensitizer (ZnPc), and Au NPs with excellent plasmonic properties, to develop a multifunctional nanotherapeutic platform with both diagnostic and therapeutic functions, termed Apt-DNA-Au nanomachines, for *in situ* imaging and targeted PDT/PTT/CDT synergistic therapy of breast cancer (Figure 2) Yu et al. (2021). Moreover, the tightly packed DNA sequences on the surface of nanomaterials were used as highly specific aptamers, which not only resisted the enzymatic hydrolysis of DNA sequences, but also improved the tumor targeting of PDT (Lv et al., 2021). Su et al. took persistent luminescent nanoparticles (PLNPs) as the core, and formed a novel nanoprobe TCPP-gDNA-Au/PLNP for persistent luminescence imaging-guided photodynamic therapy by coupling DNA sequences containing AS1411 aptamers through AuNPs Su et al. (2021). PLNPs could emit long-term fluorescence under near-infrared light irradiation. The AS1411 aptamer could specifically recognize the overexpressed nucleolin in cancer cells and improve the tumor targeting of the photosensitizer TCPP. Furthermore, increasing the types of DNA modified on the surface of NPs can improve the accuracy of PDT tumor identification. Cai et al. designed and synthesized a nano-therapeutic platform for fully automatic diagnosis and treatment of Au/Pd nanomachines with the main marker miRNA-21 and two auxiliary markers miRNA-224 and TK-1 mRNA as the targeted detection unit (Au/Pd ONP-DNA nanomachine) Cai X. et al. (2021). Using ONPs as a carrier, when all specified targets were detected [logic system input was (1, 1, 1), output was (1, 1)], the 808 nm laser could be programmed to automatically irradiate the tumor and perform PDT and PTT. However, the stability of the Au-S bond is poor, and the DNA is non-specifically detached from the surface of AuNPs, resulting in false positive signals and severe side effects. Zhang et al. used a simple and highly stable amide bond (-CO-NH-) instead of the Au-S bond and combined the DNA probe on Au@graphene (AuG) to prepare Label-rcDNA-AuG Zhang J. et al. (2021). Label-rcDNA-AuG could improve the anti-interference ability of nanoprobes against nucleases, GSH and other biological agents. By accurately monitoring the level of intracellular miR-21, Label-rcDNA-AuG could identify positive cancer cells even in a mixture of cancer cells and normal cells, improving the precision of PDT treatment and reducing damage to normal cells.

Besides DNA modification, surface modification of biotin (Bt) is also an effective way to improve the tumor targeting of NPs (Li J. et al., 2021). The targeting of Bt-modified Au-NPs (BT@Au-NPs) to C6 glioma cells was more than 2 times that of Au-NPs (He F. et al., 2021). Modification of arginine (R)-glycine (G)-aspartic acid (D) (RGD) on the surface of NPs, which could bind to integrin α_v_β_3_ integrin with high affinity, could not only improve the anticancer ability of NPs, but also reduced tumor migration rate. The HB-AuNRs@cRGD prepared by Liu et al. had a tumor inhibition rate of up to 77.04% in the ECA109 esophageal cancer model, and significantly reduced the migration and invasion of cancer cells in ECA109 cells Liu D. et al. (2021).

Besides improving the PDT efficiency of Au-NPs in combination with other nanomaterials, oxygen production can also be optimized by changing the physical structure of Au-NPs. By adjusting the ratio of silicon core radius and gold shell thickness, Sajid et al. enhanced the field strength of Au nanoshells with 40/20 core radius/shell thickness by 35 times and increased ^1^O_2_ yield by 320% compared to before optimization (Farooq and de Araujo, 2021). Furthermore, the introduction of ionic complexes assembled by heterometallic colloids (Mo_6_-Au_2_) can affect the cytotoxicity, cellular internalization and PDT activity of NPs by regulating the order of their supramolecular stacking (Kirakci et al., 2021). For example, through the 1:1 binding of [Mo_6_I_8_(L′)_6_]^2−^ (L′ = I-, CH_3_COO-) with [Au_2_L_2_]^2+^ (L was the ligand of cyclic double amines and phosphine), Faizullin et al. synthesized a heterometallic colloids composed of positively and negatively charged metal-organic complexes Faizullin et al. (2021). Among them, the cellular internalization of Mo_6_-Au_2_ (L′ = CH_3_COO-) assembled with poly-DL-lysine (PL) exhibited a three-fold enhancement when L′ = CH_3_COO- and could accumulates in the cytoplasm by fast endo-lysosomal escape. In addition, the photodynamic effect of [Mo_6_I_8_ (L′)_6_]^2−^ clusters was much higher at L′ = CH_3_COO- than at L′ = I-. In recent years, bimetallic NPs are also improving the therapeutic efficiency of PSs due to the inherent properties of the introduced metal elements and the interaction between two metal atoms (Park et al., 2021). He et al. synthesized Au_1_Bi_1_-SR NPs by introducing Bi into captopril-coated Au NPs (Au-SR NPs) by utilizing the X-ray CT signal enhancement effect of Bi He G. et al. (2021). Au_1_Bi_1_-SR NPs not only exhibited higher ROS yield than Au-SR NPs, but also enabled CT imaging-guided and light-mediated PDT for synergistic tumor therapy. In addition, Jia et al. further modified Au-Bi bimetallic nanoparticles with IR808 fuel and prepared Au-BiGSH@IR808 to obtain higher NIR photon capture ability, which effectively solved the problem of low absorption rate of Au-Bi nanoparticles in the near-infrared region Jia et al. (2021). Noble metal Pt nanozymes have catalase-like activity (Cao et al., 2021). Truncated octahedral Au (ToHAu) can enhance LSPR by increasing the spatial separation and realizing the simultaneous participation of holes and electrons in the reaction due to the special structure of twin planes and stacking faults (Yoon et al., 2017). Accordingly, Bu and his team designed and synthesized a comprehensive phototherapy nanomodulator (ToHAu@Pt-PEG-Ce6/HA) based on the enhanced LSPR effect Bu et al. (2021). Pt was deposited on ToHAu to form a spatially separated structure, which enabled the reactive molecules to freely enter the hot holes and electron fluxes, and had strong photothermal and photodynamic properties. The antitumor effect of NPs is also closely related to their size. For example, large NPs (>100 nm), despite their enhanced permeability and penetration (EPR) effect, cannot fully infiltrate into tumor tissue due to dense extracellular matrix and elevated interstitial fluid pressure (Zmerli et al., 2021). In contrast, small NPs (<20 nm) exhibited better tumor penetration but were easily cleared by the blood circulation. Therefore, it is particularly important to design a TME-responsive size-tunable NPs. To this end, Liu et al. designed and prepared Au-MB-PEG NPs by exploiting the efficient polymerization ability of AuNPs and the excellent performance of TME ROS-triggered HOCl-responsive platform Liu H. et al. (2021). Small-sized Au-MB-PEG NPs responded to highly expressed HOCl in the tumor region through a HOCl-sensitive molecule (FDOCl-24). After reaching the tumor tissue, Au-MB-PEG NPs were cleaved by HOCI to release Au NPs that rapidly aggregated into larger aggregates in the tumor through electrostatic interactions, and simultaneously released methylene blue as a photosensitizer for photodynamic therapy (PDT). The aggregated AuNPs red-shifted the light absorption to the NIR region, resulting in enhanced photoacoustic imaging (PAI) and PTT under laser irradiation.

##### 4.1.1.2 Ag Based Nanoparticles

**FIGURE 3:**
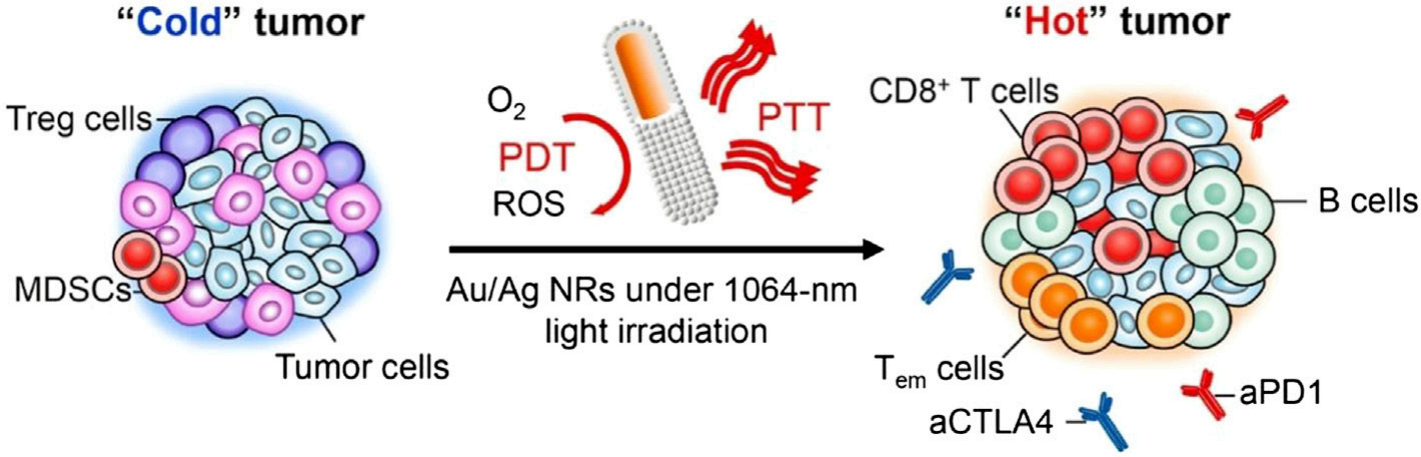
Illustration of Corn-like Au/AgNR-mediated antitumor immune responses. Corn-like Au/Ag NR-mediated NIR-II PTT/PDT significantly increased the expression of calreticulin, high-mobility group box 1, and adenosine triphosphate in tumor cells, reprogramming the immunosuppressive cold tumor microenvironment to immunogenic heat tumor, which achieve the combined anti-cancer activity with the ICB antibody and effectively inhibit the growth of distant tumors and prevent tumor recurrence. Reproduced with permission from (Jin L. et al., 2021).

Ag NPs have higher ^1^O_2_ yield than Au NPs and Pt NPs due to stronger SPR (Younis et al., 2021). Inspired by the ability of ICD to transform tumors from cold to heat by inducing tumor cells to release tumor antigens and damage-associated molecular patterns (DAMPs) and then enhance T cell proliferation and infiltration (Xu et al., 2022). Jin et al. developed a corn-like Au/Ag nanorod (Au/Ag NR) that could induce ICD in tumor cells under NIR-II (1064 nm) light irradiation (Figure 3) Jin F. et al. (2021). They covered the Ag shell on Au NRs, and by changing the amount of Ag^+^, the SPR band in the vertical region with higher ^1^O_2_ yield was red-shifted to the NIR-II window. In addition, Au/Ag NRs could maintain the immune memory effect for up to 40 days in animal experiments by enhancing the therapeutic effect of immune checkpoint blocking (ICB). Wang and his team further used DNA probe technology to prepare the DNA-functionalized nanoprobe Au-AgNP-Ag-HM loaded with the photosensitizer hematoporphyrin monomethyl ether (HMME) Wang et al. (2021b). Au-AgNP-Ag-HM not only had the ROS production mediated by the LSPR signal change of Au-Ag-HM and photosensitizer HMME, but also had fluorescent probe technology mediated by caspase-3 specific recognition sequence (DEVD), which effectively integrate the functions of pro-apoptosis and detection, and can be used for the treatment and efficacy evaluation of tumor cells.

##### 4.1.1.3 Cu Based Nanoparticles

**FIGURE 4:**
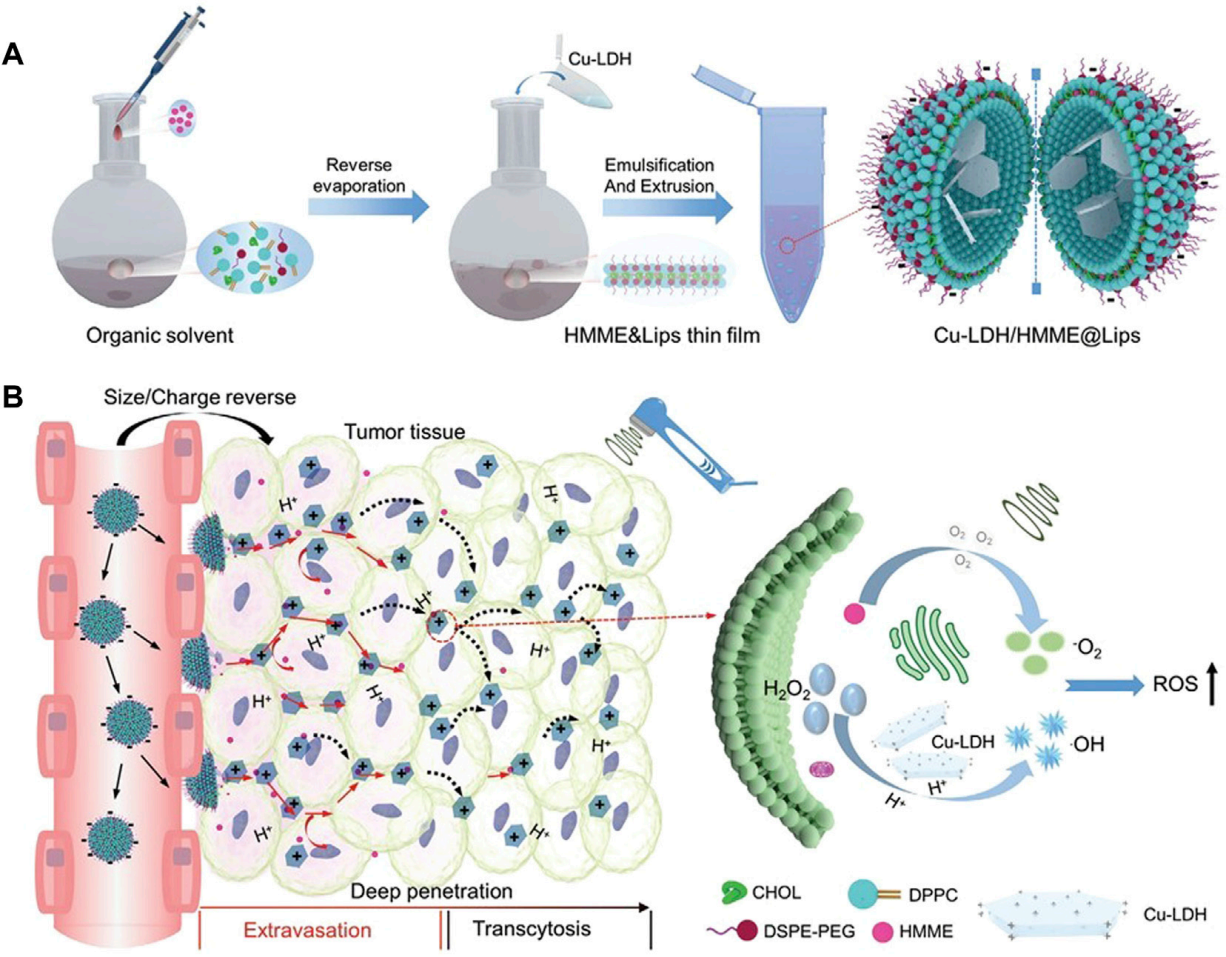
Schematic diagram of the synthesis and working principle of Cu-LDH/HMME@Lips. **(A)** Schematic diagram of the synthesis of Cu-LDH/HMME@Lips. **(B)** Schematic diagram of the working principle of Cu-LDH/HMME@Lips. Dual-size/charge-switchable Cu-LDH/HMME@Lips utilizes negatively charged liposomes to prolong circulation residence time in low-permeability solid tumor models, and then HMME decompose Cu-LDH/HMME@Lips in response to ultrasound to release positively charged Cu-LDH, which can penetrate deep into tumor cells. HMM generates ^1^O_2_ under ultrasound irradiation, while Cu-LDH infiltrates deep in tumor generates ROS through a Fenton-like reaction. Reproduced with permission from (Wu et al., 2021).

Cu-based Fenton reagents have superior ROS yields to Fe-based systems (Zhu F. et al., 2021). In addition, Cu-doped layered double hydroxide (Cu-LDH) nanosheets can not only further enhance the yield of ROS, but also take advantage of the small size and positive charge to actively infiltrate cancer cells for deep tumor therapy (Wu et al., 2021). But positively charged NPs have a shorter residence time in the blood circulation than negatively charged NPs (Smith et al., 2019). To this end, Wu and his team used negatively charged liposomes to encapsulate Cu-LDH (Cu-LDH@Lips) and embedded HMME into the bilayer of CuLDH@Lips, forming a dual size/charge switchable reactive oxygen species generator (Cu-LDH/HMME@Lips) (Figure 4) Wu et al. (2021). Liposomes could prolong the residence time in the circulatory system by reducing the clearance of Cu-LDH/HMME@Lips by immune cells, and HMME could disintegrate Cu-LDH/HMME@Lips in response to ultrasound to release positively charged Cu-LDH, which could penetrate deep into tumor cells and then caused oxidative stress damage to tumor cells through Fenton-like reaction. Similarly, Wang et al. prepared ICG/CAC-LDH nanosheets by intercalating indocyanine green (ICG) into a hydrophobic bilayer of Ce-doped Cu-Al layered double hydroxide (CAC-LDH) Wang X. et al. (2021). ICG/CAC-LDH could not only induce the depletion of intracellular GSH, but also decompose to generate Cu^+^ and Ce^3+^ to stimulate the Fenton-like reaction to generate OH.

##### 4.1.1.4 Ruthenium Based Nanoparticles

Ru complexes are commonly used as PDT PSs due to their high water solubility, photostability, and high ROS yield (Smithen et al., 2020). However, it has the disadvantages of dark toxicity, DNA mutation and being excited only by short-wave visible light, which limit its clinical application (Chen M. et al., 2021). To this end, He et al. synthesized a new red light-responsive Ru complex PSs (Ru-I) without two-photon activation, by using large conjugated indolepyridine benzopyrans as ligands He Y. et al. (2021). Positively charged Ru-I could effectively target cancer cell lysosomes and activate PSs under 660 nm red light to induce apoptosis. In addition, Karges et al. proposed to wrap Ru (II) polypyridine complexes with amphiphilic polymer DSPE-PEG_2000_-folate to prepare nanoparticles without dark toxicity, which could target cancer cells overexpressing the folate receptor Karges et al. (2021). It exhibited significant phototoxicity under irradiation at 480 or 595 nm and induced tumor cell apoptosis through the caspase3/7 pathway. Furthermore, this complex not only shown the highest 1- and 2-photon absorption to date, but also exhibited properties of inhibiting multidrug-resistant tumors in a rat model. In addition to the introduction of folate groups, bio-orthogonal labels such as copper-catalyzed azide-alkyne cycloaddition (CuAAC) are also effective methods to improve tumor cell recognition (Kappenberg et al., 2022). Lin et al. prepared the first bio-orthogonal two-photon PSs based on Ru (II) complexes by bio-orthogonally labeling Ru-alkynyl-2 Lin C.-C. et al. (2021). In addition to generating ROS to exert cytotoxic effects, it could also exert anti-tumor effects by specifically binding to cancer cell membranes and inducing membrane damage.

##### 4.1.1.5 Iridium Based Nanoparticles

**FIGURE 5:**
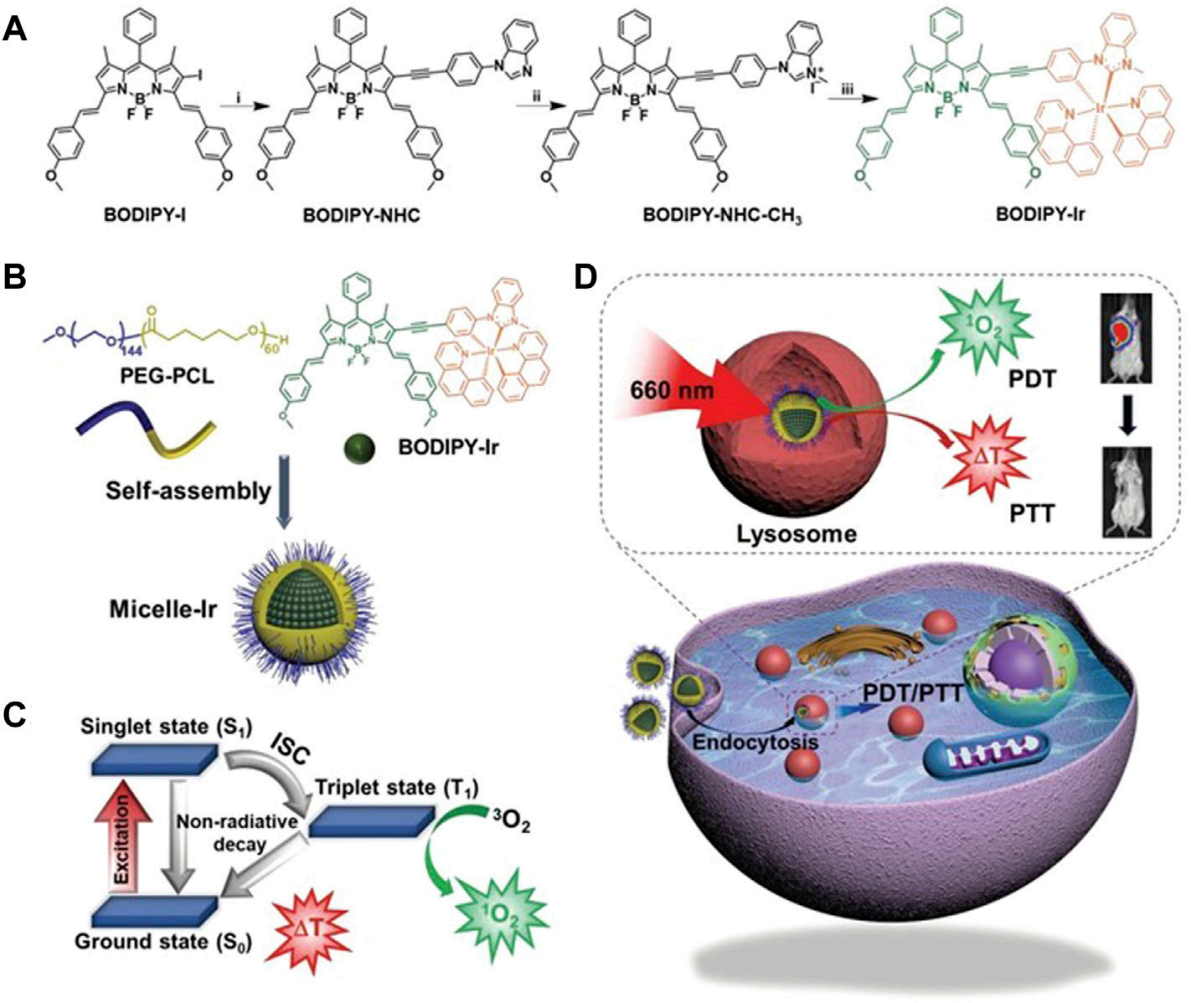
Schematic diagram of synthesis of BODIPY-IR and schematic diagram of PDT/PTT synergistic therapy mechanism. **(A)** Schematic synthesis of BODIPY-IR: Reagents and conditions: 1) 1-(4-ethynylphenyl)-1H-benzo [d]imidazole, CuI, Pd (PPh3)4, THF, TEA, 50°C, 16 h; 2) MeI, THF, 100°C, 24 h; 3) [Ir (benzo [h]quinoline)2 (µ-Cl)]2, Ag2O, 1,2-dichloroethane, reflux, 24 h. **(B–D)** Schematic representations of Encapsulation of BODIPY-Ir into micelles for constructing Micelle-Ir. **(B)** PEG-PCL-encapsulated BODIPY-Ir self-assembles into Micelle-Ir. **(C)** Photophysical processes of PDT and PTT. **(D)** Michelle-Ir-mediated PDT/PTT synergistic therapy *in vivo*. Reproduced with permission from (Liu S. et al., 2021).

In recent years, new photoredox catalyst systems based on Ir compounds have been widely used in catalysis and PDT, and can also be used to prepare new PSs (Raza et al., 2021). Liu and his team prepared two Ir (III) complex dimers that could self-assemble into NPs in aqueous media, named Ir1 and Ir2 Liu J. et al. (2021). Ir1 and Ir2 not only enhance cancer cell uptake through positive charges on the surface, but also exhibit type I and type II PDT activity in the 350–500 nm (UV-Vis spectrum) range, even in hypoxic microenvironments. Ir NPs might be promising alternatives to traditional organic PSs. Ir (III) complexes show great potential in the construction of oxygen-sensitive sensing probes due to their unique oxygen quenching pathway (Yasukagawa et al., 2021). Xiao et al. designed and synthesized a red light-excited Ir (III) complex encapsulated in the hydrophobic pocket of Cyanine7-modified β-cyclodextrin (β-CD) Xiao and Yu (2021). Ir (III) complexes achieved different degrees of oxygen quenching according to the change of oxygen concentration in the environment. β-CD could not only be used to improve the water solubility of Ir (III) complexes, but also be used to carry Cyanine7 to establish a proportional oxygen fluorescent probe. The results of *in vitro* and *in vivo* experiments shown that the prepared probe had remarkable oxygen sensitivity and could be used for quantitative determination of the oxygen level in the hypoxic microenvironment of solid tumors. Mitochondria are not only the decisive regulators of cellular metabolic function and apoptosis, but also the organelles with the highest intracellular oxygen concentration (Tabish and Narayan, 2021). Therefore, the preparation of PSs that target the mitochondria of cancer cells will be the key to maximize the therapeutic potential of PDT. Recently, Redrado et al. prepared four novel mitochondria-selective trackable PSs, which are called bifunctional Ir (III) complexes of the type [Ir (CˆN)_2_ (NˆN-R)]+, where NˆC is either phenylpyridine (PPY) or benzoquinoline (BZQ), NˆN is 2,20-dipyridylamine (Dpa), and R either anthracene (1 and 3) or acridine (2 and 4) (Redrado et al., 2021). Only complex 4 ([Ir (Bzq)_2_ (dpa-acr)]+) clearly shown a dual emission mode. The organic luminescent chromophore (acridine) could display the cellular localization of the complexes under irradiation at 407–450 nm. Ir (III) had more than 110-fold higher photosensitivity values under 521–547 nm irradiation than under dark conditions, and promoted apoptotic cell death and a possible apoptotic pathway by generating ROS. Although two-photon near-infrared photoactivation can partially overcome the lack of absorption or weak absorption of Ir (III) complexes in the red to near-infrared region, the requirements of ultrafast femtosecond laser source and small irradiation area limits its application in solid tumor therapy. To this end, Liu and his team designed and synthesized bifunctional micelles (Micelle-Ir) for synergistic PDT and PTT therapy *in vivo* (Figure 5) Liu N. et al. (2021). They were prepared by micellization of a neutral Ir (III) complex (BODIPY-Ir) containing a distyryl boron-dipyrrole methylene group (BODIPY-Ir). BODIPY enabled BODIPY-Ir to acquire the ability to absorb in the far-red/near-infrared region, and micellization enabled BODIPY-Ir to acquire the ability to synergize PDT and PTT therapy, not only destroying primary 4T1-Luc tumors, but also preventing lung metastases.

#### 4.1.2 Metal Oxide-Based Nanoparticles

Metal oxide nanomaterials have found promising biomedical applications for fluorescent labeling due to the advantages of high photostability, large extinction coefficient, high emission quantum yield and easy surface modification (Younis et al., 2021). In view of the fact that nano-heterostructures can promote photo-induced electron-hole separation and the generation of ROS, 2D nano-heterostructure-based PSs can provide a major advancement in PDT. Qiu et al. designed and synthesized a bismuthene/bismuth oxide (Bi/BiO_x_)-based lateral nano-heterostructure synthesized by a regioselective oxidation process Qiu et al. (2021). Upon irradiation at 660 nm, the heterostructure could effectively generates ^1^O_2_ under normal oxygen conditions but produces cytotoxicity OH and H_2_ under hypoxic conditions, which synergistically improves the intensity of PDT. In addition, this Bi/biox nano-heterostructure had biocompatibility and biodegradability, with the surface molecular engineering used here, it improved the penetrability of tumor tissue and increased the cellar uptake, and then produced an excellent oxygen-independent tumor ablation effect. Iridium dioxide (IrO_2_) with semiconductor behavior has high catalytic activity for oxygen release reaction (OER) in a wide pH range, and also has excellent photocatalytic efficiency (Arias-Egido et al., 2021). For this, Yuan et al. synthesized IrO_2_-Gox@HA NPs that could target TME by combining glucose oxidase (GOx) and IrO_2_ NPs on hyaluronic acid (HA) Yuan Y. et al. (2022). First, Gox converted the high levels of glucose in tumors to H_2_O_2_, and then IrO_2_ NPs converted H_2_O_2_ to O_2_, thereby enhancing type II PDT, which could effectively alleviate hypoxia in tumor tissues.

Relevant literature points out that the larger the specific surface area of the sheet-like structure, the higher the zeta potential, and the greater the drug-carrying capacity of nanomaterials (Duo et al., 2021). Dai et al. synthesized a radiosensitizer based on 4-layer O-Ti_7_O_13_ nanosheets by using two-dimensional titanium peroxide nanomaterials with mesoporous structure to support DOX Dai Y. et al. (2021). O-Ti_7_O_13_ could quickly load the drug within 5 min while other nanomaterials need 24 h and release the drug continuously in an acidic microenvironment. In addition, under the action of high-energy X-rays, titanium dioxide could absorb radiation energy to synthesize ROS and kill tumor cells. O-Ti_7_O_13_ could reduce the X-ray dose and irradiation frequency, and reduce damage to normal tissues while ensuring the therapeutic effect. Mn^3+^-rich oxides (MnO_x_) can be decomposed into catalytically competent Mn^3+^ and Mn^2+^ in TME, accelerating the conversion of endogenous O_2_ into highly toxic ^1^O_2_ and OH. Meanwhile, the released Mn^2+^ can be used as a magnetic resonance imaging agent with higher spatial resolution. Studies have shown that Zinc gallogermanate (ZGGO) persistent luminescent NPs can be loaded with therapeutic drugs to achieve long-term imaging tracking of drugs and significant tumor treatment effects. To improve the poor resolution of ZGGO, Ding et al. used MnO_x_ to coat chromium-doped ZGGO NPs (Mn-ZGGO) Ding D. et al. (2021). Mn-ZGGO could not only generate ^1^O_2_ and OH without light, but also serve as a diagnostic tool for MR, US, and sustained luminescence to guide precise cancer therapy. Similarly, Li and his team used MnO_2_ and IrO_2_ to prepare nanozymes (MnO_2_/IrO_2_-PVP, MIP NPs) to combine photothermal effect, catalytic effect and magnetic resonance imaging function Li R. et al. (2021). IrO_2_ enabled the photothermal conversion efficiency of MIP NPs to reach 27.57%. Polyvinylpyrrolidone (PVP) enabled the Ce6 loading efficiency of MIP NPs to be as high as 76.07 ± 0.52%, and could shed PVP from the surface of MIP NPs in an acidic microenvironment, realizing the local enrichment of MIP NPs at tumor. Besides MnO_2_, CeO_2_ also has similar functions to catalase (CAT) and superoxide dismutase (Yang Y.-L. et al., 2021). Zeng et al. prepared a dual-targeted tumor drug delivery system (ICG@PEI−PBA−HA/CeO_2_) by using ICG, inorganic nanozyme (HA/CeO_2_) and pH-sensitive cationic polymer carrier phenylboronic acid-modified polyethyleneimine-4-carboxyphenylboronate (PEI-PBA) Zeng et al. (2021). After ICG@PEI−PBA−HA/CeO_2_ targeted cancer cells with HA, the CeO_2_ released from the pH cleavage reaction of phenylboronic acid catalyzeed H_2_O_2_ to generate O_2_ through the cerium valence cycle of Ce^3+^/Ce^4+^. The regenerable CAT-like nanozyme activity of CeO_2_ increased the bioavailability of ICG and promoted tumor cell apoptosis by improving the tumor hypoxic microenvironment.

The efficacy of PDT/PTT combination therapy was better than that of PDT or PTT monotherapy. Traditional PDT/PTT synergistic therapy requires two light sources to excite the PSs of PDT and the thermosensitive agent of PTT respectively, which increases the difficulty of nanoparticle preparation. Guo and his team used B-TiO_2_ with oxygen vacancies and narrow band gaps to prepare a nanothermosensitive system (B-TiO_2_@SiO_2_-HA) with full-spectrum response to light stimulation Guo et al. (2021). Under NIR-II laser irradiation, B-TiO_2_@SiO_2_-HA could not only provide PDT/PTT synergistic therapy for tumors, but also perform high-resolution photoacoustic imaging (PAI) to achieve precise nanothermothermal effects. Similarly, Gao and his team used SnO_2-x_ with oxygen vacancies to prepare a multifunctional nano-thermosensitive material (SnO_2-x_@SiO_2_-HA) with a target-specific synergistic PDT/PTT with full spectrum response Gao et al. (2021a). In addition to exerting precise PDT/PTT synergistic antitumor therapeutic effect through PAI, SnO_2-x_@SiO_2_-HA also had antibacterial effect, which effectively promotes the healing of skin wounds. But most metal or carbon NPs are toxic to normal cells or tissues. Sengupta et al. used the biosafety and magnetic properties of magnetite (Fe_3_O_4_) nanoparticles to synthesize a new PS E-NP with anti-inflammatory and immunoprotective effects Sengupta et al. (2022). E-NP could not only upregulate the expression of cyclin kinase inhibitory protein p21, but also inhibit cancer cell cycle arrest in sub-G0G1 phase. It also acted as an anti-inflammatory by reducing macrophage myeloperoxidase (MPO) and nitric oxide (NO) release, thereby minimizing collateral damage to healthy cells.

#### 4.1.3 Upconversion Nanoparticles

**FIGURE 6:**
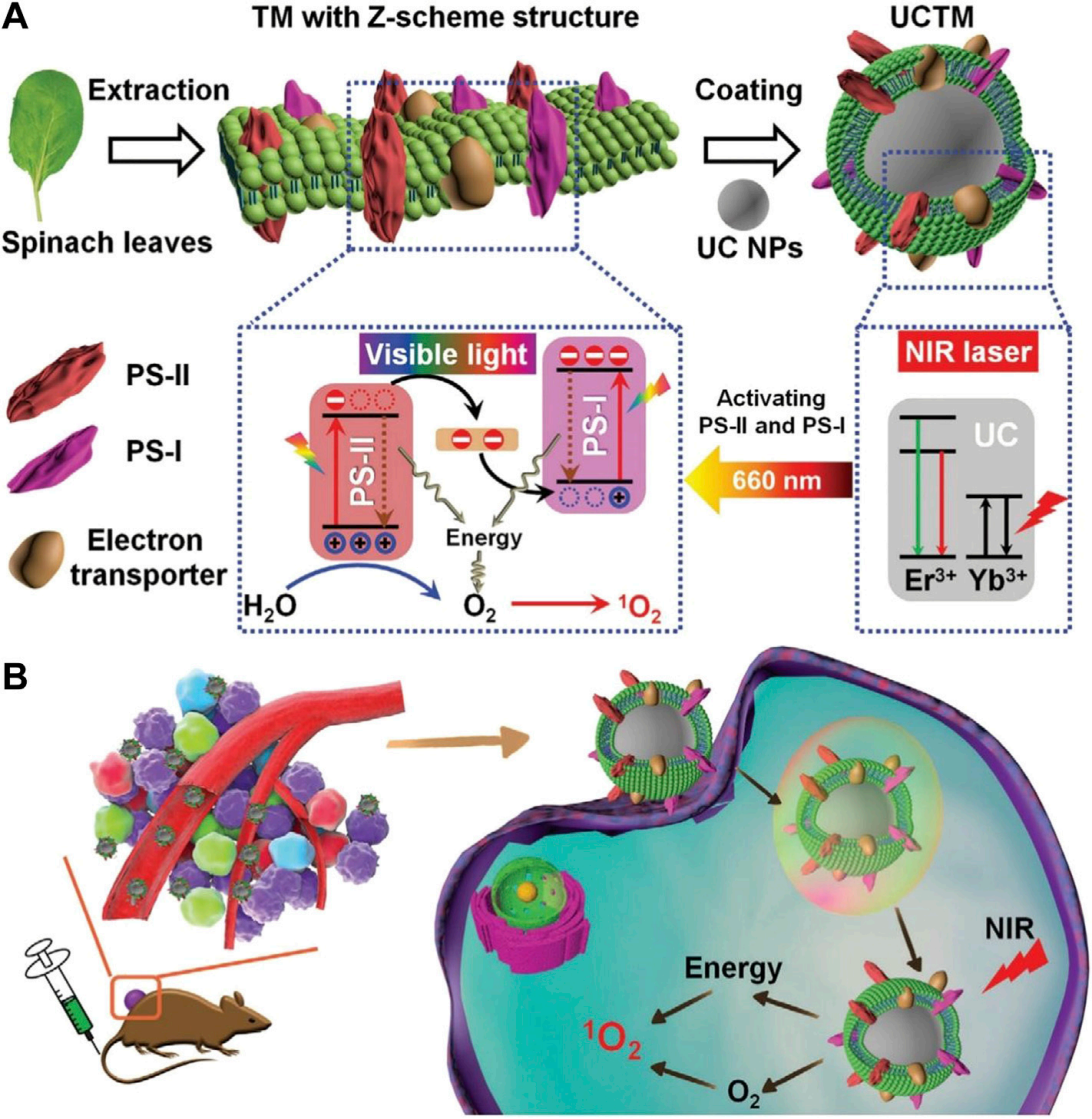
Schematic illustration of the PDT effect of UCTM NPs on hypoxic TME. **(A)** The fabrication process of UCTM NPs. The corresponding electron transfer of UCTM NPs under NIR laser irradiation and the mechanism for realizing spatiotemporally synchronized O_2_ self-supply and ROS production. **(B)** The therapeutic process of oxygen supply and ROS generation by UCTM NPs under NIR laser irradiation. Reproduced with permission from (Cheng Y. et al., 2021).

UCNPs have nonlinear anti-Stokes properties and can emit high-energy photons under low-energy NIR light excitation through lanthanide ion doping. UCNPs also have the advantages of low toxicity, narrow emission bandwidth, large decay time, resistance to photobleaching, and no autofluorescence background (Liu et al., 2022). The emission wavelength of UCNPs can be controllably adjusted from ultraviolet light to near-infrared light to match PSs with different absorption wavelengths, which provides a new method to solve the problem of PDT light penetration depth (Zhang L. et al., 2021). The photosystem-I/photosystem-II (PS-I/PS-II) PDT system can only be excited by red light to generate O_2_, so that it can quickly supply its own O_2_ consumption in ^1^O_2_ production, showing a spatiotemporal synchronous system of O_2_ self-supply and ROS production. However, the tissue penetration ability of red light is unsatisfactory, so it is unsuitable for the removal of deep tissue tumors (Fakurnejad et al., 2019). Recently, Cheng et al. used the ability of UCNPs can emit red light to activate PS-I and PS-II under NIR light, decorated thylakoid membrane of chloroplasts on UCNPs to form UCTM NPs, and developed a new photosynthesis-based PDT strategy for realizing spatiotemporally synchronous O_2_ self-supply and ROS production (Figure 6) Cheng X. et al. (2021). Both *in vitro* and *in vivo* assessments prove that UCTM NPs can effectively relieve hypoxia, induce cell apoptosis, and eliminate tumors with NIR light irradiation. A large amount of APT produced during the ICD process can be hydrolyzed by the extracellular enzyme CD73 into ADO (immunosuppressant), which prevents the cytotoxic T-cell immune response (Workenhe et al., 2021). To this end, Jin and his team prepared cancer-cell-biomimetic UCNPs (CM@UCNP-Rb/PTD) by exploiting the properties of anti-CD73 antibody to block the adenosine pathway Jin F. et al. (2021). CM@UCNP-Rb/PTD utilized the cancer cell membrane (CM) to target cancer cells and avoid macrophage uptake. After reaching the tumor tissue, UCNPs converted NIR into visible light to generate ROS, and released DOX to achieve a Chemo-PDT synergistic combination therapy. CD73-blocked CM@UCNP-Rb/PTD enhanced spontaneous antitumor immunity through the combined effect of chemotherapeutic drugs, PDT-triggered ICD and CD73 blockade. In immunotherapy, ligands that block PD-L1 can also directly prevent PD-1/PD-L1 immune blockade (Lee et al., 2021). Liu and his team modified UCNPs with MIPs formed by Pd-L1 peptide phase transfer imprinting, and prepared MC540/MNPs@MIPs/UCNP composite imprinted particles Lin M. et al. (2021). MC540/MNPs@MIPs/UCNP utilized MIPs to target tumor cells, which improved the binding rate to tumor cells and achieved targeted PDT. The nucleus is the control center of cellular biological activities, and targeting PDT to the nucleus can lead to severe DNA damage and inactivation of nuclear enzymes (Teng et al., 2021). Base on this, Chen and his team proposed a PDT strategy of “one treatment, multiple irradiation” Chen X. et al. (2021). They modified hollow mesoporous silica nanoparticles with amine group with acidification effect and loaded RB and UCNPs to synthesize UCNP/RB@mSiO_2_-NH based on the lysosome-nucleus pathway. After UCNP/RB@mSiO_2_-NH entered the lysosome, it entered the nucleus through the nuclear pore by generating ROS and destroying the lysosome under the first 980 nm (3 min) NIR irradiation. Subsequently, efficient nuclear-targeted PDT was achieved under a second 980 nm NIR irradiation. Dual PSs PDT is also one of the effective strategies to improve ROS yield. Pham and his team utilized SiO_2_-coated core-shell UCNPs to support two PSs (Rb and Ce6) to form UCNP/RB, Ce6 Pham et al. (2021). UCNP/RB, Ce6 produced a large amount of ^1^O_2_ under 1550 nm NIR-IIb irradiation, which had higher PDT efficiency than single PS. However, the application of UCNPs in the biomedical field still faces challenges due to the low quantum yield and superheating effect of the 980 nm light source, and the low drug loading capacity (Lee et al., 2020).

#### 4.1.4 Carbon-Based Nanoparticles

However, g-C_3_N_4_NSs have poor PDT efficacy due to wide band gap and low utilization of visible light (He F. et al., 2021). Graphitic carbon nitride nanosheets (g-C_3_N_4_NSs) are a recently reported promising carbon-based nanomaterial with the advantages of high biocompatibility, high photoluminescence quantum yield and surface modifiability. However, g-C_3_N_4_NSs have poor PDT efficacy due to wide band gap and low utilization of visible light (Younis et al., 2021). It has been proposed that the PDT efficiency of g-C_3_N_4_NSs can be enhanced by doping metal ions or UCNPs.

Although Ru (II) polypyridine complexes have high ^1^O_2_ generation and stability, the hypoxic microenvironment limits their PDT efficacy (Karges et al., 2020). Therefore, Wei’s group synthesized a novel oxygen self-sufficient PS (Ru-g-C_3_N_4_) by grafting [Ru (Bpy)_2_]^2+^ onto g-C_3_N_4_ nanosheets through Ru-N bonds Wei et al. (2021). The incorporation of [Ru (Bpy)_2_]^2+^ enabled Ru-g-C_3_N_4_ to obtain high loading capacity, narrow bandgap and high stability, thereby greatly improving the PDT efficiency. In addition, Ru-g-C_3_N_4_ not only had catalase-like activity in hypoxic environment, but also could effectively react with H_2_O_2_ to generate free radicals, causing oxidative stress damage to tumor cells. Molybdenum carbide (MoxC) has an electronic structure similar to that of noble metals, among which, Mo_2_C nanospheres can simultaneously induce PTT and PDT under illumination in the entire near-infrared radiation region due to their metallic properties and interband/intraband transitions, exhibiting highly efficient redox capacity (Zhang et al., 2019). Based on this, Hou and his team prepared a novel nanocomposite, Mo_2_C@N-Carbon-3@PEG, by combining Mo_2_C nanospheres with N-carbon. N-carbon could improve the photogenerated charge separation of Mo_2_C@N-Carbon-3@PEG, which doubles its photocatalytic performance Hou et al. (2022). However, the doping of metal ions and integration with other nanomaterials increases the risk of biological toxicity and side effects on the one hand, and increases the complexity of the PSs multistep synthetic protocols on the other hand. Based on this, Liu’s group had designed and synthesized a nitrogen-rich graphitic carbon nitride nanomaterial, 3-amino-1,2,4-triazole (3-AT) derived g-C_3_N_5_NSs Liu X. et al. (2021). The photocatalytic activity of g-C_3_N_5_NSs was 9.5 times higher than that of g-C_3_N_4_NSs. Compared with g-C_3_N_4_, the nitrogen-rich triazole group could achieve low-energy transition by reducing the band gap of g-C3N5NSs conjugation, thereby improving the utilization efficiency of visible light.

#### 4.1.5 Sulfur-Based Nanoparticles

Metal sulfides are often used as photothermal agents for photothermal therapy, among which Ni_3_S_2_, CuS and Co_3_S_4_ nanoparticles can be used to synthesize semiconductor PSs, which is an effective way to combine PTT/CDT/PDT (Shukla et al., 2021).

Co_3_S_4_ NPs will be degraded in the acidic microenvironment and trigger a Fenton-like reaction to generate hydroxyl radicals (·OH), which exerts the efficacy of CDT. Based on this, Jiang and his group prepared a nanocomposite Co_3_S_4_-ICG, which could responsively release PSs in an acidic microenvironment and realize the synergistic antitumor effect of CDT/PTT/PDT under NIR by loading indocyanine green (ICG) into hollow Co_3_S_4_ Jiang Y. et al. (2021). The combination of Fenton-like reaction and PDT enhanced ROS production and antitumor effect. Similarly, Feng and his team designed and prepared a nanocomposite FeS_2_@SRF@BSA with bovine serum albumin (BSA) as a carrier to encapsulate FeS_2_ NPs and SRF, for combining CDT, PTT and PDT to achieve trimodal synergistic tumor therapy Feng et al. (2021). In addition, the Z-scheme heterostructure possesses both narrow band gap and highly oxidative holes, which are helpful for inducing near-infrared photocatalytic oxygen production (Mafa et al., 2021). Based on this, Sang’s group had designed and synthesized a Z-scheme nanoheterostructures, Ni_3_S_2_/Cu_1.8_S@HA Sang et al. (2021). The doping of Cu improves the near-infrared absorption and photothermal conversion efficiency by 7.7% compared with Ni_3_S_2_. It not only had catalase/peroxidase-like activity to generate endogenous O_2_ to relieve hypoxic internal environment, but also had a targeted recognition effect on cancer cells with high CD44 receptor expression, showing good anti-cancer properties effect.

#### 4.1.6 Phosphorus-Based Nanoparticles

Black phosphorus (BP) is a kind of post-graphene two-dimensional (2D) nanomaterials with special structural in-plane anisotropy, which have better optical and electrical properties than carbon-based metal NPSs and sulfur-based metal NPSs (Younis et al., 2021). In recent years, microorganisms with photosynthetic properties and biocompatibility, such as cyanobacteria, have been rapidly developed and used in cancer therapy and other diseases related to oxygen tension (Dahiya et al., 2021; Qamar et al., 2021). Qi and his team hybridized cyanobacterial cells with 2D BP nanosheets to form a microbe-based nanoplatform, Cyan@BPNSs Qi et al. (2021). *In vivo* experiments, Cyan@BPNSs could effectively increase the oxygenation level in the tumor and maintain a tumor inhibition rate of more than 100%.

Although black phosphorus quantum dots (BPQDs) have larger surface area and higher ^1^O_2_ quantum yield than BP (up to 0.91 in oxygen-saturated solution), the instability and poor tumor targeting of BPQDs in physiological environment limit their further research and clinical applications (Ding S. et al., 2021; Liu Y. et al., 2021). To this end, Liu and his team used the cationic polymer polyethyleneimine (PEI) to modify BPQDs, and modified RGD peptides targeting tumor cells on their surfaces to prepare BPQDs@PEI + RGD-PEG + DMMA, which had pH-responsive charge-switching and tumor-targeting properties Liu et al. (2021i). The results shown that BPQDs@PEI + RGD-PEG + DMMA had good stability *in vivo*, and could increase the uptake of BPQDs by tumor cells through charge conversion under light, and achieved tumor target enrichment. Most tumor microenvironment-responsive nanoparticles improve the targeting of nanomaterials by responding to the hypoxic microenvironment, but many tumor tissues do not exhibit hypoxia (Huang C. et al., 2020). The hypoxic sites of tumors are mostly located deep in the core of metabolically active tumor tissues, but traditional nanomaterials have the disadvantage of weak tissue penetration (Chen et al., 2019). To this end, Ding and his team were the first to combine BPQDs and genetically engineered *E. coli* expressing catalase by electrostatic adsorption to form hybrid engineered *E. coli*/BPQDs (EB) Ding D. et al. (2021). EB could dissolve the cell membrane of *E. coli* carrying catalase under light exposure, and then the released catalase could generate oxygen to improve hypoxia in the tumor.

Besides BP, red phosphorus (RP) can also generate reactive oxygen species under visible light irradiation (Zhang P. et al., 2021). Among them, Z-type RP/BP nanosheets prepared by exploiting the high separation efficiency of electron-hole pairs not only have photocatalytic activity, but also ROS can be generated with higher efficiency (Liu et al., 2019). To this end, Kang and his team prepared M-RP/BP@ZnFe_2_O_4_ NSs hybrid nanomaterials with higher stability and tumor targeting by loading Z-type RP/BP nanosheets with ZnFe_2_O_4_ and tumor cell membranes Kang et al. (2022). Among them, ZnFe_2_O_4_ could not only increase the productivity of ROS by catalyzing the Fenton reaction, but also induced apoptosis of MB-231 cells through oxidative stress.

#### 4.1.7 Metal-Organic Frameworks

MOFs are a new class of molecular crystal materials, composed of metal ions or clusters bridged by organic connectors. MOFs can integrate NPs and/or biomolecules into a single framework hierarchically through taking advantage of their synthetic tunability and structural regularity, and can be used as highly active sites for multifunctional therapy (Gao et al., 2021b). Focusing on the key factors of O_2_ production, Ren et al. designed and assembled a novel two-stage intelligent oxygen generation nanoplatform based on metal organic framework core modified by Pt and CaO_2_ NPs (UIO@Ca-Pt) Ren et al. (2021). It was based on the porphyrin metal-organic framework (UIO), and at the same time loaded by CaO_2_ NPs with polydopamine (PDA), and then used Pt to further improve biocompatibility and efficiency. In TME, CaO_2_ could react with water to increase the content of H_2_O_2_. These H_2_O_2_ were further decomposed into O_2_ by Pt NPs, thereby promoting the TCPP in the nanometer parent nucleus to convert the surrounding O_2_ into ^1^O_2_ under laser irradiation. On the other hand, as the most toxic ROS,·OH has stronger oxidizing property and better therapeutic properties than ^1^O_2_ (Manivasagan et al., 2022). Recently, the strategy to induce *in situ* generation of OH has been proposed by introducing Fenton-based agents into tumor cells to induce over-expressed H_2_O_2_ in tumor cells. Chen et al.’s research focused on designing nanocarriers for the transport of Fenton catalysts or metal ions such as iron ions. They synthesized MIL-101(Fe)@TCPP using nanoscale iron-based metal-organic framework MIL-101(Fe) loaded with 5,10,15,20-tetrakis (4-carboxyphenyl) porphyrin (TCPP) PS Chen et al. (2021d). MIL-101(Fe) catalyzed the conversion of H_2_O_2_ to OH through Fenton reaction under acidic TME, and served as a nanocarrier to deliver TCPP PS to generate photoactivated ^1^O_2_ for tumor-specific therapy without serious side effects, showing antitumor great potential for applications.

Iron-based MOFs have the advantages of high drug loading capacity, adjustable degradability and flexible structure. Porphyrin-based MOFs can be used as PSs for high-efficiency PDT with high stability (Ren et al., 2021). Hypoxic TME and endogenous antioxidant defense (AOD) (such as high expression of glutathione and GSH) can weaken the therapeutic effect of PDT. Therefore, breaking cellular redox balance through ROS enrichment and AOD inactivation may lead to effective tumor suppression with high clinical significance (Zhao et al., 2020). By combining iron with porphyrin-based MOFs, the maximum anti-tumor efficacy can be exerted by intelligently responding to exogenous and endogenous stimuli. Inspired by all this, Yu et al. first prepared a metal organic framework nanosystem (NMOF) based on coordination between Fe (III) and TCPP by a one-pot method (Fe-TCPP NMOF). Then, after capping the surface of silk fibroin (SF) to form NMOF@SF NPs, this nanoplatform can load a hypoxia-activated precursor tirapazamine (TPZ) to form NMOF@SF/TPZ (NST) Yu et al. (2022). Utilizing Fe (III) in Fe-TCPP, NST could effectively react with tumorous GSH to generate glutathione disulfide (GSSG) and Fe (II), for the ineffectiveness of AOD system. On the other hand, Fenton-like activity of Fe (II) and TCPP-mediated PDT promoted the accumulation of OH and ROS, and aggravated the intracellular oxidative stress under light laser irradiation. The redox metabolism disorder caused by the ineffective AOD and the enrichment of ROS might cause irreversible tumor cell damage. In addition, the deoxygenation of PDT led to an increase in hypoxia and then activated TPZ to transform into cytotoxic benzotriazinyl (BTZ) for tumor-specific chemotherapy. NST could achieve complete tumor elimination *in vitro* and *in vivo*. Similarly, Wang et al. also prepared nano-carrier system Zr-MOF@PPa/AF@PEG based on the consideration that PDT oxygen consumption could activate chemotherapy (CT) drugs to solve the hypoxic challenge and improve anti-tumor effect Wang Y. et al. (2021). Nano-carrier system Zr-MOF@PPa/AF@PEG included zirconium ion metal organic framework (UIO-66, carrier), pyropheophorbide-a (PPa, PS) and 6-amino flavone (AF, hypoxic-sensitive drug). Under ultraviolet light, although Zr-MOF@PPa/AF@PEG accepted part energy of PPa, leading to the ^1^O_2_ yield (40%) was lower than that of PPa (60%), it solved the problem of self-agglomeration caused by hydrophobicity of PPa, thereby improving the PDT effect. In addition, Zr-MOF@PPa/AF@PEG produced ^1^O_2_ under light stimulation with temporal-spatial selectivity. Therefore, Zr-MOF@PPa/AF@PEG took advantage of the PDT-induced hypoxia to activate HIF-1 inhibitor AF to enhance the anti-tumor effect and achieve the synergistic PDT-chemotherapy (PDT-CT) therapeutic effects. In addition, tuning the PDT efficiency by adjusting the thickness of the MOF shell is also a good approach. The Au@MOF core-shell hybrids prepared by Cai et al. could tune the thickness of the MOF shell by controlling the interlayer coordination reaction Cai Z. et al. (2021). As the MOF shell thickness increased, the percentage of TCPP and the efficiency of PDT also increased.

**FIGURE 7:**
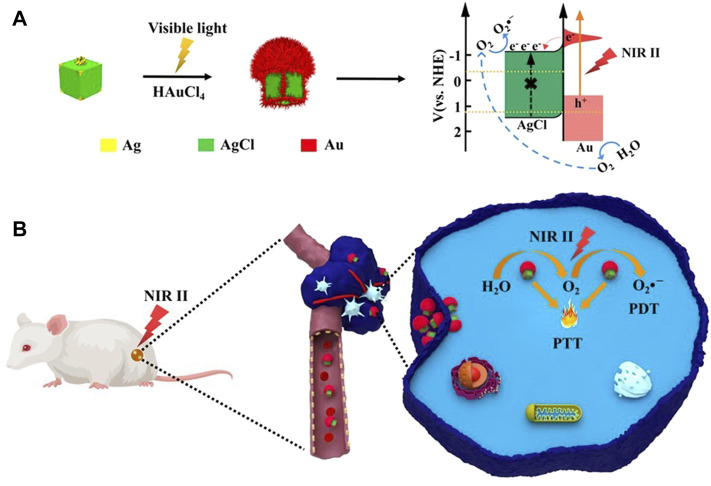
Synthesis of Ag-AgCl@Au NMs and schematic diagram of PDT/PTT synergistic therapy under NIR-II irradiation. **(A)** Schematic diagram of the synthesis of Ag-AgCl@Au NMs. Schematic diagram and electron energy level diagram of O_2_ reacting with electrons in AgCl nuclear conduction band to generate superoxide anion radical (O_2_^-^). **(B)** Schematic diagram of PDT/PTT synergistic therapy under *in vivo* NIR-II light irradiation of Ag-AgCl@Au NMs. Under NIR-II light irradiation, the plasmonic effect of Au nanostructures generates O_2_ through photocatalysis and enhances the yield of O_2_^−^. Reproduced with permission from (Liu D. et al., 2021).

The use of solar energy to drive the photocatalytic reaction has always been considered an ideal way to obtain O_2_ (Liu et al., 2021j). Compared with the previous oxygen-generating substances, water-splitting materials have the unique advantage of the O_2_ self-supply and generation of ROS, because there is abundant water in the organism. At present, ultraviolet (UV) light, visible light (Nguyen et al., 2021) and a limited region of the first near-infrared (NIR-I) (650–950 nm) light (Huang L. et al., 2020) can activate water-splitting materials to achieve light-driven endogenous water oxidation (photocatalytic water-splitting reaction) to obtain O_2_ and ROS, but water splitting materials with NIR-II light triggered molecular O_2_ generators have not been reported for tumor therapeutics, even though NIR-II can provide deeper tissue penetration. For this, Liu et al. synthesized a new type of plasmonic Ag-AgCl@Au core-shell nanomushrooms (NMs) by selectively photodepositing plasmonic Au at the bulge sites of the Ag-AgCl nanocubes (NCs) (Figure 7) Liu B. et al. (2021). Under NIR-II light irradiation, the plasma effect of the Au nanostructure could overcome hypoxia to oxidize endogenous H_2_O to produce O_2_, thereby alleviating the hypoxic microenvironment. Almost at the same time, O_2_ could react with the electrons on the AgCl nuclear conduction band to generate superoxide anion radicals (O_2_^−^) for photodynamic therapy. In addition, the combination index value of PDT for Ag-AgCl@Au NMs was 0.92, indicating that Ag-AgCl@Au NMs with excellent PDT properties toward further and promoting the PDT effect in deep O_2_-deprived tumor tissues. It is also a research hotspot in recent years to use the functionalization of NMOFs with stimuli-responsive gating units to develop signal-controlled drug delivery systems for biomedical applications. One important subcategory of gated drug-loaded NMOF includes stimulus-responsive nucleic acid-locked drug-loaded NMOF, gaining from the remarkable versatility of nucleic acid sequences to generate recognition elements and structural elements (Zhao et al., 2020). For this, Zhang et al. designed and synthesized the UIO-66 metal-organic framework nanoparticles (NMOF) modified with aptamer-functionalized DNA tetrahedra functionalized by ATP-aptamer or VEGF-aptamer could be loaded with the DOX, which responded to ATP or VEGF to release the drug Zhang S. et al. (2021). They utilized VEGF-responsive tetrahedral-gated NMOFs to load the photosensitizer Zn (II) protoporphyrin IX (Zn (II)-PPIX) to synthesize Zn (II)-PPIX/G-quadruplex VEGF aptamer-tetrahedral nanostructures. VEGF triggered the release of Zn (II)-PPIX from the complex. Association of the released Zn (II)-PPIX with the G-quadruplex structure yielded a highly fluorescent supramolecular Zn (II)-PPIX/G-quadruplex VEGF aptamer-tetrahedral structure, enabling efficient PDT treatment of malignant cells.

### 4.2 Nanoliposomes

Nanoliposomes are single-lamellar or multilamellar nano-systems formed spontaneously when phospholipids are dispersed in aqueous medium. Except biocompatibility and biodegradability, liposomes have high structural flexibility, which can combine a variety of hydrophilic and hydrophobic drugs to improv their solubility and pharmacokinetics (Cheng X. et al., 2021). In addition, liposome nanotechnology can also co-package a variety of PSs and/or drugs, provide sufficient binding sites for conjugation with a variety of functional ligands.

**FIGURE 8:**
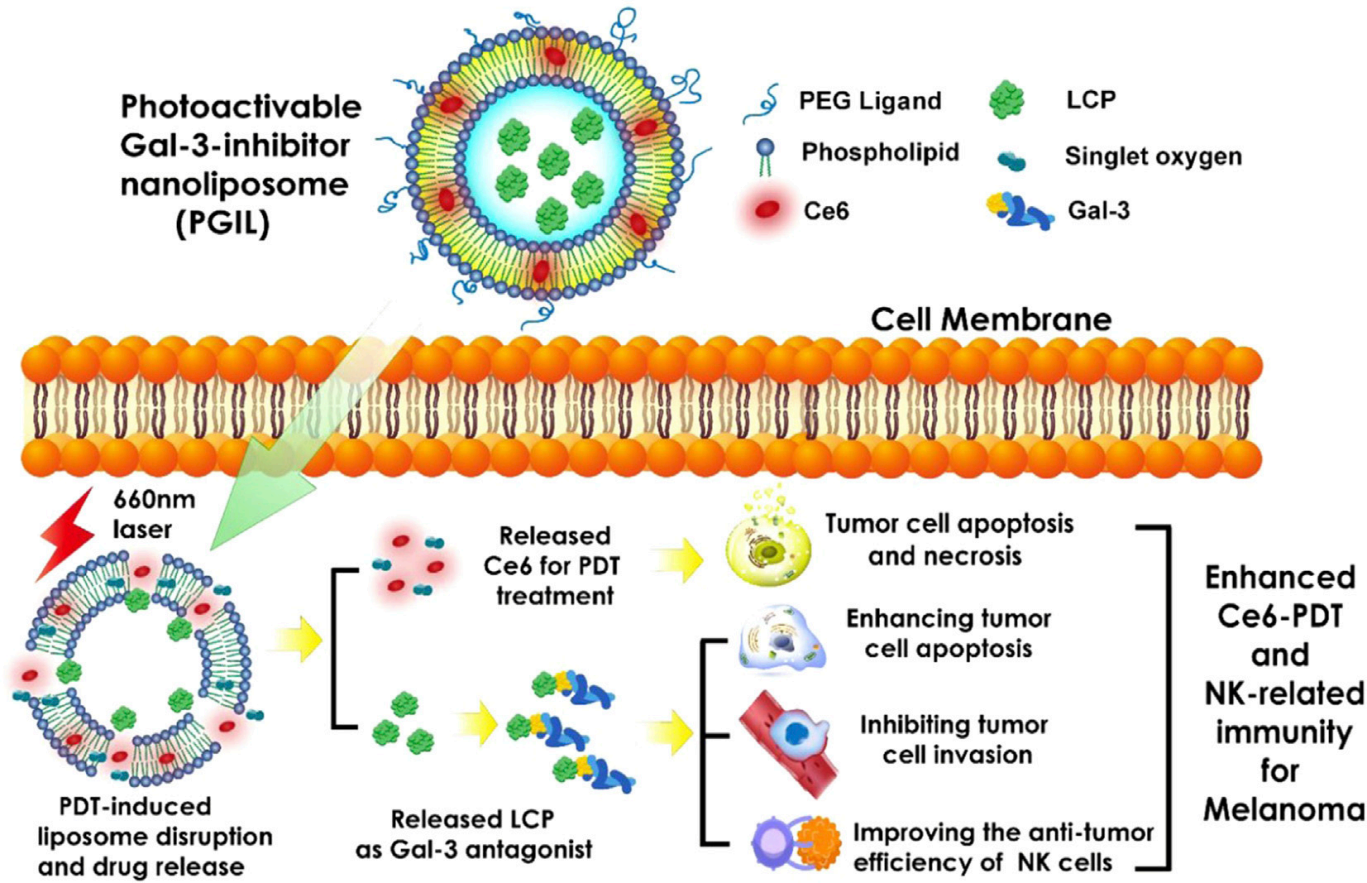
Structure of prostacyclin and its sensitizing effect in tumor cells. After PGIL is taken up by cancer cells, ^1^O_2_ generated by Ce6 triggered by NIR causes the liposomes in the outer layer to rupture, releasing Ce6 and LCP. Ce6 mediates PDT, while LCP enhances PDT-induced apoptosis, inhibits cell metastasis, and activates NK cell-mediated immune clearance by inhibiting galectin-3. Abbreviations: Gal-3, galectin-3 protein. Reproduced with permission from (Wang et al., 2019).

Several mechanisms have demonstrated that high concentrations of soluble NKG2DLs derived from tumor cells may inhibit tumor immunity and NK cell-mediated target cell lysis by downregulating the expression of NKG2DL, thereby contributing to tumor immune escape (Curio et al., 2021). Based on this, Wang’s group had designed and synthesized a Chlorin-based photoactivable Galectin-3-inhibitor nanoliposome (PGIL), which could combine photosensitizer chlorin e6 (Ce6) and low molecular citrus pectin (LCP) (Figure 8) Wang et al. (2019). The intracellular release of LCP inhibits the activity of galectin-3, which increases the affinity of major histocompatibility complex (MHC) proteins on tumor cell membrane for NKG2D on NK cell membranand, and then increases the tumor cell apoptosis, inhibits the invade ability, and enhances the recognition ability of NK cells to tumor cells in melanoma cells after PDT. In addition, pharmacological inhibition of MMPs reduce the level of released NKG2DLs, which could increase the tumor cell surface expression of NKG2DLs, reverse their immunosurveillance escape properties, and make them easier to be cleared by immune cells (mainly NK cells) (Tampa et al., 2021). Based on this, Liu’s group had designed and synthesized a PS-MMP inhibitor nanoliposome (i.e., Ce6-SB3CT@Liposome [Lip-SC]), which combines the PS, Ce6, and a matrix metalloproteinase (MMP) inhibitor (i.e., SB3CT) Liu H. et al. (2021). Nanoliposomes have significant anti-tumor proliferation and metastasis efficacy after laser irradiation in A375 cells. The relatively fast internalization of Lip-SC could accumulate in the tumor area under 660 nm light irradiation, induce apoptosis in cancer cells, which could trigger an immune response. In addition, it could also induce the expression of NK group 2 member D ligand (NKG2DL), while activate NKG2D, thus, NK cells could better recognize and kill tumor cells. The subsequent release of SB-3CT could further activate NK cells effectively and strengthen the immune system though inhibiting the shedding of soluble NKG2D ligands. As a result, Lip-SC caused induce apoptosis in cancer cells regardless of the presence or absence of irradiation. In the process of PDT agents inducing tumor cell necrosis and apoptosis, local inflammation occur and a variety of tumor cell antigens are exposed, thereby inducing local immune responses. Therefore, by supplementing immunomodulators to enhance immune activity, the anti-tumor effect of PDT can be increased. Dual-ligand-modified NPs increased the number of total liposomes bound to cancer cells through dual-ligand modification, and had higher tumor enrichment capacity and PDT efficiency than single-ligand-modified NPs. For example, Li and his team prepared Fru-Bio-Lip by co-modifying liposomes with fructose (targeting fructose transporter) and biotin (targeting multivitamin transporter) ligands Li J. et al. (2021). Besides targeted aptamer modification, the combination with near-infrared light-activated photon thermodynamic therapy (PTDT), PTT and other phototherapy methods is also an effective strategy to improve the tumor killing effect of PDT. Dai et al. synthesized thiadiazoloquinoxaline semiconductor polymer (PTT), 2, 2′-azobis [2-(2-imidazolin-2-yl) propane] dihydrochloride (AIPH) (PTDT prodrug) and γ-aminoacetic acid (GA, heat shock protein inhibitor) were incorporated into thermosensitive liposomes, which were then modified with targeting aptamers to form Lip(PTQ/GA/AIPH) Dai Z. et al. (2021). Under NIR-II laser irradiation, Lip (PTQ/GA/AIPH) could achieve precise diagnosis and effective suppression of deep triple-negative breast cancer. However, the clinical application of nanoliposomes in PDT still faces challenges due to poor stability *in vivo* and low drug loading (Cheng Y. et al., 2021).

### 4.3 Mesoporous Silica Nanoparticles

**FIGURE 9:**
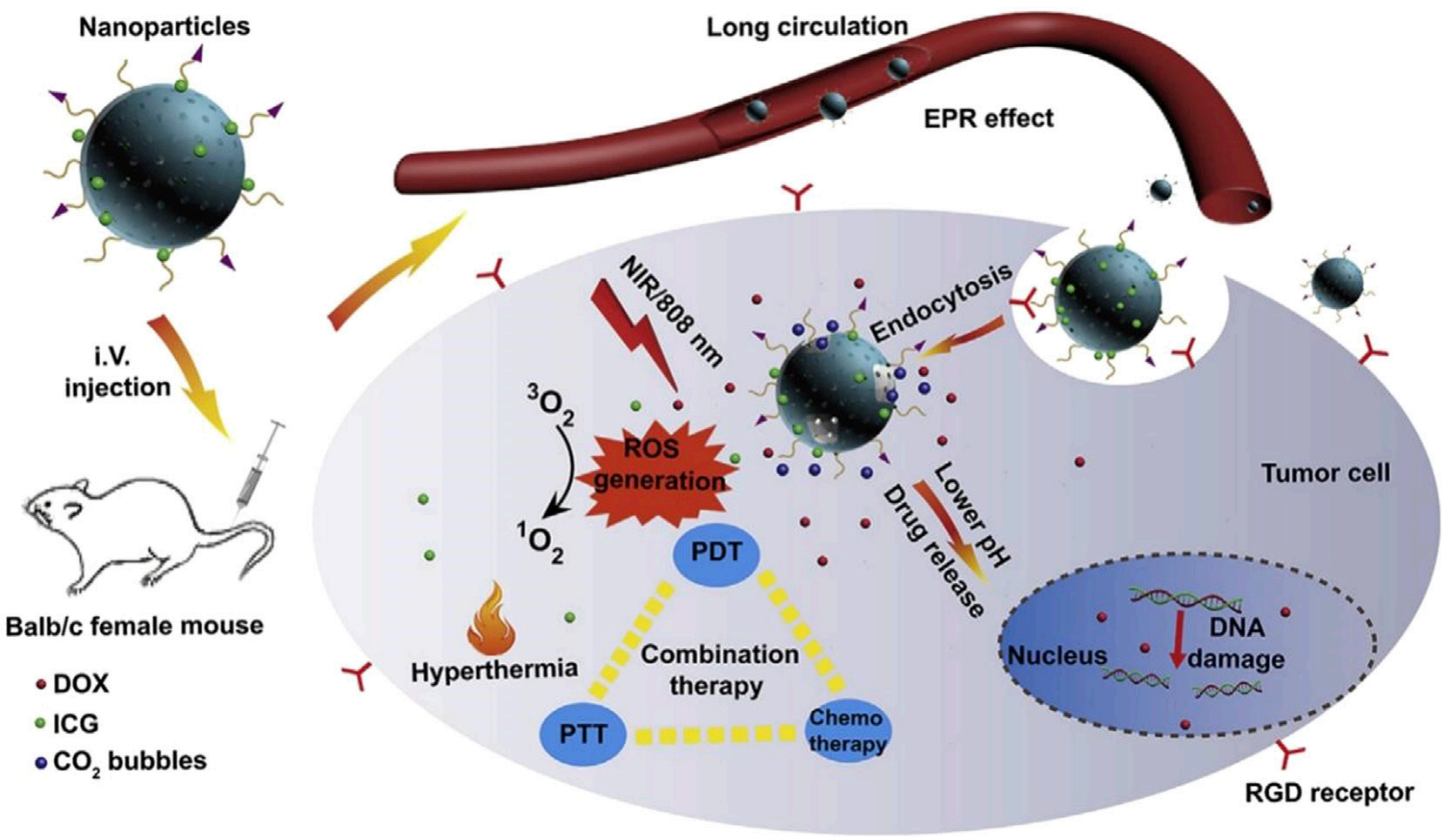
Schematic illustration of M (A)D@PI-PEG-RGD for enhanced tumor combination therapy. M(A)D@PI-PEG-RGD targets tumor cells through RGD, generates ROS and induces temperature increase through ICG under NIR irradiation. In addition, the acidic microenvironment accelerates the release of DOX by breaking down NH_4_HCO_3_, which combine chemotherapy, PTT and PDT to enhance the therapeutic effect. Reproduced with permission from (Zhang J. et al., 2021).

Among various nanomaterials, mesoporous silica nanoparticles (MSNs) have become an important nano-delivery system for PDT and multiple combination therapy due to their unique physical and chemical advantages, such as high loading capacity, controllable pore size and morphology, versatile surface chemistry, satisfying biocompatibility and biodegradability, which can ensure stable and efficient loading of PSs, targeted delivery of PSs, and regulation of drug release and cellular uptake behavior (Wang Z. et al., 2021). Hypoxia is a typical feature of tumor TME, which seriously affects the efficacy of PDT. The development of nano-enzymes with the ability to produce oxygen is a promising strategy to overcome the oxygen-dependent of PDT. In this regard, Chen’s group had designed and synthesized a dual-nanozymes based cascade reactor HMSN@Au@MnO_2_-Fluorescein Derivative (HAMF), which was composed of hollow mesoporous silica nanoparticles (HMSN), high-efficiency photosensitizer 4-DCF-MPYM (4-FM), ultra-small Au-NPs and MnO_2_ Chen et al. (2021e). 4-Fm was a thermally activated delayed fluorescence (TADF) fluorescein derivative with high fluorescence quantum yield, photostability, two-photon excitation and low biological toxicity. Au NPs exhibited glucose oxidase (GOx)-mimic ability that could catalyze glucose into gluconic acid and H_2_O_2_, simultaneously, the consumption of glucose could cut off the energy supply of tumor cells (Zhang Y. et al., 2021). With the response to the hypoxic microenvironment, MnO_2_ could catalyze H_2_O_2_ into O_2_ and accelerate the oxidation of glucose by Au NPs to generate additional H_2_O_2_, which was used as a substrate for the catalytic reaction of MnO_2_, which can be used in light irradiation, thereby constantly producing ^1^O_2_ for enhanced PDT upon light irradiation. HAMF could alleviate tumor hypoxia and achieve an effective tumor inhibition *in vitro* and *in vivo* studies. Yin’s team had also designed and synthesized a kind of H_2_O_2_-responsive and oxygen-producing nanozyme by loading a large number of gold nanoclusters (AuNCs) into MSNs to form nanoassembly, and wrapped MnO_2_ nanosheets in the form of a switch shielding shell (denoted as AuNCs@mSiO_2_@MnO_2_) Yin et al. (2021). In a neutral physiological environment, stable MnO_2_ shells could switch off PDT by eliminating the generation of ^1^O_2_. However, in an acidic TME, the MnO_2_ shell reacted with H_2_O_2_, and simultaneously sufficient O_2_ generation guaranteed a 74% high ^1^O_2_ yield, which showing strong PDT performance. In addition, multifunctional NPs loading with chemotherapeutic drugs and PSs are also a promising method for effective tumor combination therapy. Zhang’s group had designed and synthesized a novel pH-sensitive and bubble-generating mesoporous silica-based drug delivery system (denoted as M (A) D@PI-PEG-RGD) (Figure 9) Zhang Z. et al. (2021). DOX and NH_4_HCO_3_ were loaded into MSNs pores, MSNs was coated with polydopamine (PDA) layer, and then ICG as a photothermal and photodynamic agent was loaded onto the PDA layer surface, finally the nanoparticles were modified with polyethylene glycol (PEG) and RGD. RGD is a ligand for the recognition site of integrins and displays great adhesion capacity between extracellular matrix cells and cells, which can improve the accuracy of M (A)D@PI-PEG-RGD. Under NIR irradiation, M (A)D@PI-PEG-RGD could generate ROS and induce the temperature rise performed by ICG. In addition, acidic environment and high temperature would also decompose NH_4_HCO_3_, thereby accelerating the release of DOX. In summary, the multifunctional pH-sensitive and bubble-producing M (A)D@PI-PEG-RGD combines chemotherapy, PTT and PDT to improve the therapeutic effect of tumors.

### 4.4 Dendrimers

**FIGURE 10:**
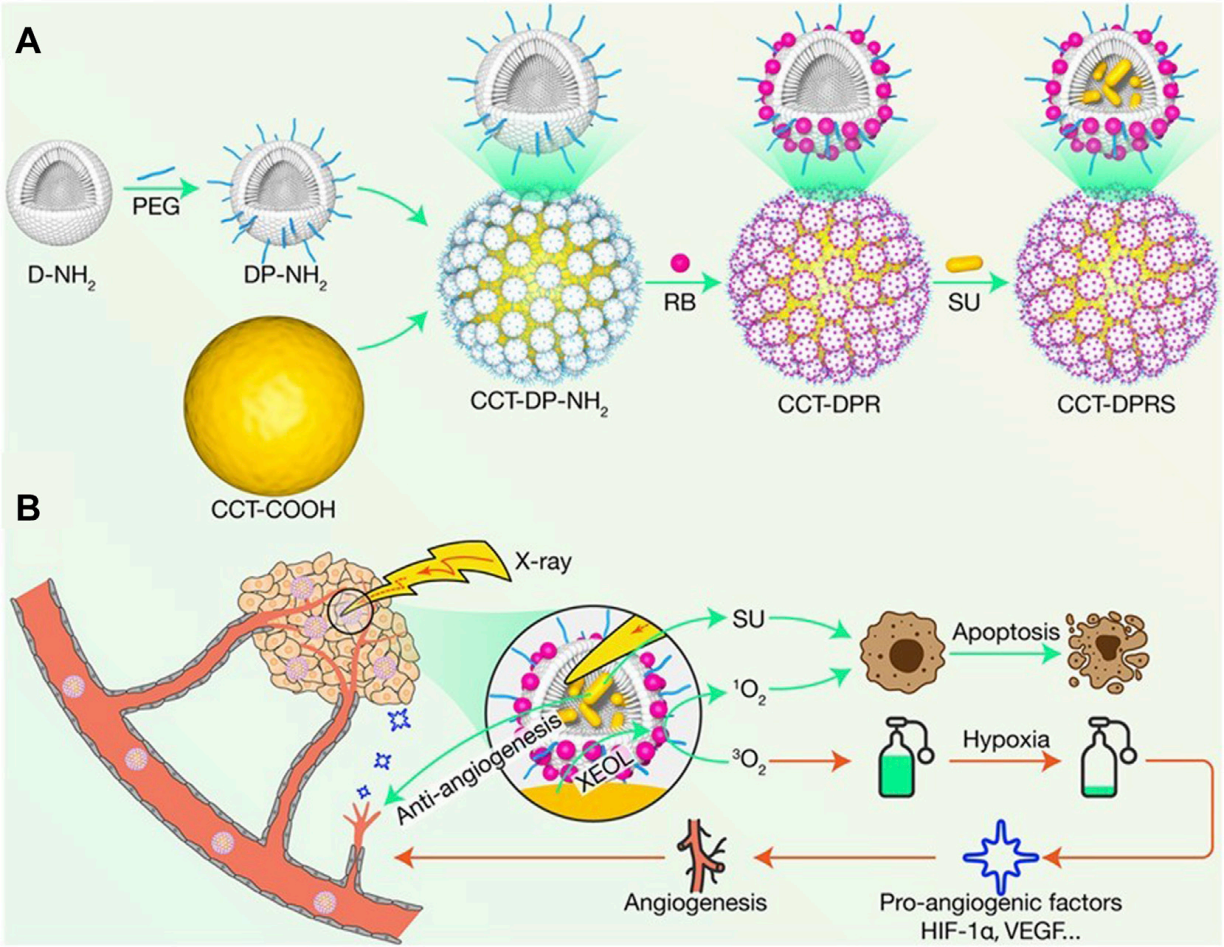
Schematic diagram of the construction and function of CCT-DPRS. **(A)** CCT-DPRS is prepared using PAMAM as an intermediate framework to load nanoscintillator, PSs, and SU. **(B)** Mechanism of combined therapy of XPDT and SU. After exposure to ultra-low dose radiation, Rb produces ^1^O_2_ to kill malignant tumor cells. Meanwhile, SU effectively block XPDT-mediated hypoxia-exacerbated tumor angiogenesis, with a clear synergistic effect. Reproduced with permission from (Jiang Y. et al., 2021).

Dendrimers are synthetic high molecular polymers with tree-like and highly branched structure. They are composed of the small initial core, the internal cavity formed by multiple branches, and the peripheral functional groups. Due to their special structure, dendrimers have the advantages of easy surface modification with PSs along with other functional moieties and structural configuration with other components or nanoformulations *via* the hyperbranched units for enhanced tumor accumulation and penetration (Ouyang et al., 2021). The combination of molecular targeted therapy of epidermal growth factor receptor tyrosine kinase inhibitors (EGFR-TKIs) and photodynamic therapy PDT can combat non-small cell lung cancer (NSCLC) with effective synergistic results (Qiao et al., 2021). However, the hypoxic TME not only affects the efficacy of PDT, but also induces EGFR-TKIs resistance. In this regard, Zhu and researchers had designed a nanocomplex APFHG by loading gefitinib (Gef) and PS hematoporphyrin (Hp) into an aptamer modified fluorinated dendrimer (APF) Zhu L. et al. (2021). Due to the targeting effect of EGFR-TKIs and the good oxygen-carrying capacity of APF, APFHG could specifically recognize EGFR-positive NSCLC cells and release Gef and Hp in response to the hypoxic acidic microenvironment. Under laser irradiation, APFHG could significantly increase the production of intracellular ROS, effectively improve the tumor hypoxia microenvironment, and overcome hypoxia-related drug resistance. In other work, X-ray-induced photodynamic therapy (XPDT) is overwhelmingly superior in treating deep-seated cancers (Chuang et al., 2020). However, low energy transfer efficiency of the therapeutic nanoplatform and hypoxic environment presented in the tumor tissue limits the therapeutic effect of XPDT. In order to improve the therapeutic effect of XPDT in deep-seated cancers, it is necessary to develop a delicate architecture to support the organization of nanoscintillator and multiple agents to improve the effectiveness of XPDT (Ahmad et al., 2019). In this regard, Zhao and researchers developed a dual-core-satellite architecture nanosmart system (CCT-DPRS) that the polyamidoamine (PAMAM) dendrimer would be used as an intermediate framework with nanoscintillator, PSs, and sunitinib (SU) (Figure 10) (Jiang Z. et al., 2021). It had high XPDT and antiangiogenetic capabilities by systematic optimizing the scintillation efficiency and nanoplatform structure. After exposure to ultralow dose radiation, the codoped CaF_2_NPs converted the trapped energy into green emission, which enable further excitation of Rb to produce ^1^O_2_ to kill malignant tumor cells. At the same time, the antiangiogenic drug SU effectively blocked tumor vascularization aggravated by XPDT-mediated hypoxia, rendering a pronounced synergy effect. However, PAMAM dendrimer and indocyanine green (ICG) have inevitable interaction with proteins and cells, which induces biological toxicity and reduces therapeutic efficacy *in vivo* (Mindt et al., 2018). To overcome these shortcomings, Cui and researchers had designed a new drug delivery system G5MEK7C (n)-ICG with a “stealth” layer Cui et al. (2021). The surface of G5MEK7C (n)-ICG was modified with p (EK) peptide, which was double-layer super hydrophilic zwitterionic material. When the pH was lower than 6.5, the surface of G5MEK7C (n)-ICG showed a positive charge, which made it more likely to interact with the cell membrane in the tumor tissue. Therefore, under laser irradiation *in vitro* and *in vivo*, due to the good targeting effect of G5MEK7C(70)-ICG, G5MEK7C(70)-ICG was more effective in killing tumors than free ICG, while the damage to the liver was less than free ICG. The combination of chemotherapy and other therapeutic modalities can overcome chemoresistance through different mechanisms of action to achieve the purpose of enhancing anti-tumor efficacy. Furthermore, adding chemicals during mitosis to block cell division may be a promising approach to promote nuclear uptake of PSs. Recently, based on dendrimers and phenylboronic acid-sialic acid interactions, Zhong and researchers modified phenylboronic acid (PBA) into the surface of dendrimers, which can selectively recognize the sialic acids, meanwhile, conjugated lipoic acid modified Ps onto the core of dendrimer Zhong D. et al. (2021). In this work, a novel tumor targeting and penetrating, GSH/ROS heterogeneity responsive and PTX-loaded dendrimeric nanoparticles (P-NPs) was developed for mutually synergetic chemo-photodynamic therapy of PTX-resistant tumors. Lentinan was coated on the periphery of dendrimers through boronate bonds, which could avoid non-specific binding of P-NPs with normal cells during blood circulation. P-NPs penetrated into tumor tissues and actively entered into the cells through the PBA-SA interactions, showing enhanced cellular uptake and tumor penetration. Subsequently, P-NPs released PTX in response to high concentrations of glutathione and H_2_O_2_ in tumor cells, arresting the cells in the G2/M phase and exerting anti-tumor effects. At the same time, the time of nuclear membrane disintegration increased caused the enhanced intranuclear photosensitizer accumulation, thereby increasing the efficiency of PDT by increasing nuclear DNA damage.

### 4.5 Hydrogels

**FIGURE 11:**
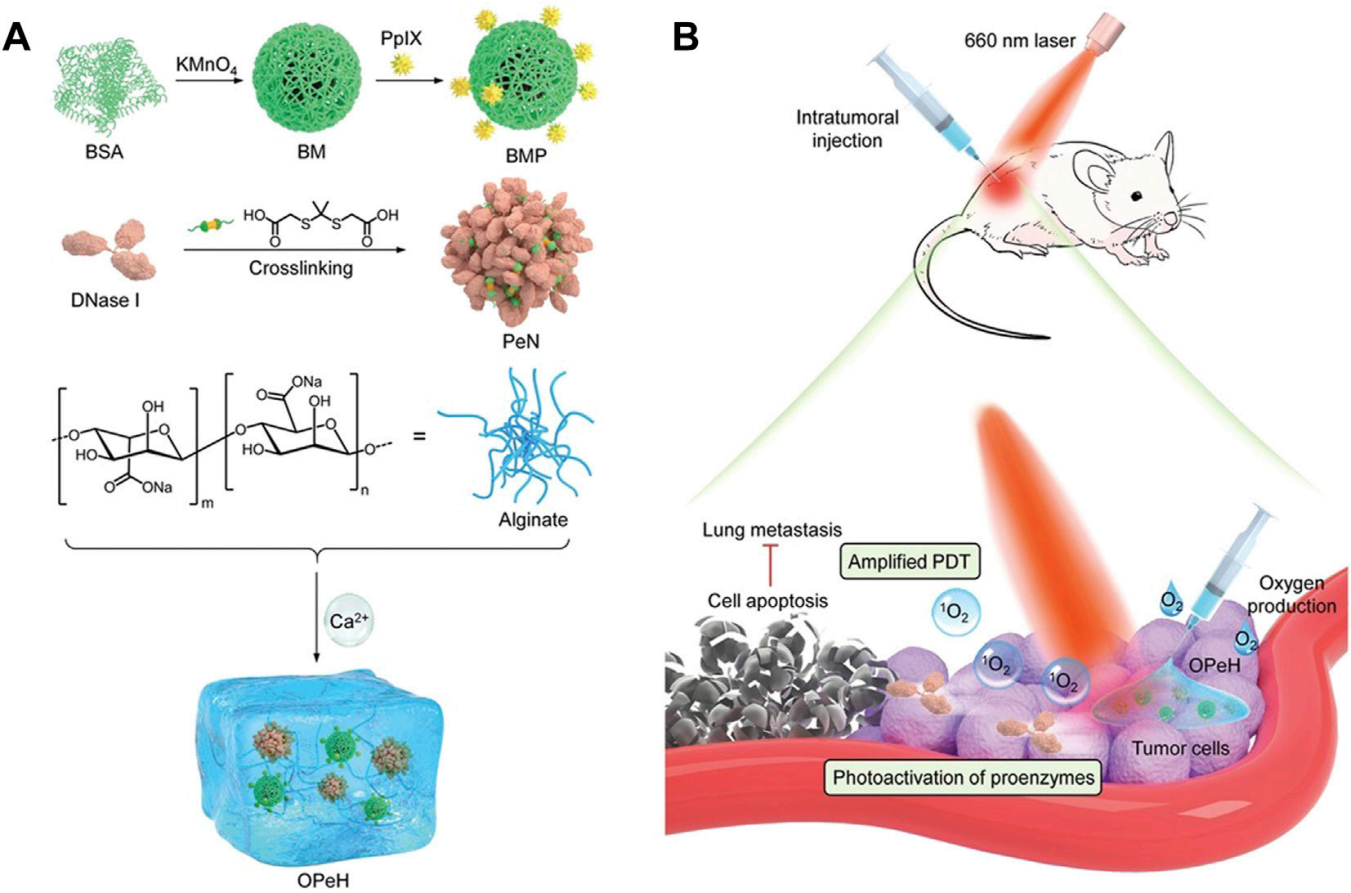
Schematic diagram of the preparation and treatment principle of OPeH. **(A)** Schematic diagram of the preparation of OPeH. **(B)** Mechanism of oxygen production and NIR photoactivation of OPeH. OPeH integrates PDT and NIR light-activated enzymes to achieve the combined therapeutic effect of inhibiting tumor growth and lung metastasis. Reproduced with permission from (Liu N. et al., 2021).

Hydrogel is a type of nano-delivery carrier system formed by a hydrophilic polymer material with a three-dimensional network structure through chemical cross-linking or physical cross-linking. Hydrogels are biocompatible and have similar physical properties like living tissues. Some drugs are easily dispersed in the hydrogel matrix (Hu et al., 2022). Therefore, hydrogels have been widely used for the delivery of hydrophilic drugs. Among them, local injection of hydrogels has received special attention in tumor treatment and prevention of tumor recurrence, because this injection method can achieve desired drug accumulation in tumors. Compared with oxygen-generating nanomaterials, prodrugs that can be activated by external light show the unique advantages of highly consistent responsiveness and high temporal and spatial selectivity (Rapp and DeForest, 2021). Recently, Liu’s group constructed a kind of original oxygen-generating hydrogel (OPeH) with photoactivated enzyme activity by loading the oxygen-generating MnO_2_ nanoparticles conjugated with protoporphyrin IXpt (PPIX), and the proenzyme nanoparticles (PeN) crosslinked by a ^1^O_2_ cleavable linker into alginate hydrogels (Figure 11) Liu J. et al. (2021). Under NIR laser irradiation, MnO_2_ NPs converted H_2_O_2_ into O_2_, which further promoted the production of ^1^O_2_ from PpIX and improved the efficiency of ^1^O_2_ generation. In addition, after PEN was cross-linked with the ^1^O_2_ cleavable linker, it induced cell death and suppressed metastasis by inhibiting the extracellular trap (NET) of neutrophils. In animal experiments, it was found that OPeH integrates PDT and NIR light-activated enzymes to achieve the combined therapeutic effect of inhibiting tumor growth and lung metastasis. In addition, phototherapy against deep tumors may greatly limited by the lack of light flux and the chemotherapy drugs against tumor cells may limited by insufficient resident time. Therefore, Zhong’s group developed a dual drug carrying system DOX-CA4P@Gel, which achieved the best curative effect, efficacy and safety through local sequential delivery of drugs Zhong Y. et al. (2021). With dextran oxide, chitosan, porphyrin and hollow mesoporous silica (HMSN), DOX-CA4P@Gel was constructed, in which combretastatin A4 phosphate (CA4P) and DOX were both loaded. In weakly acidic condition, the degradation rate of the hydrogels increased significantly. CA4P was released rapidly at the early stage and relatively stable after 48, while DOX released slowly at first and then quickly released after 48 h, showing an obvious sequential release behavior. The porphyrin in hydrogel could trigger the formation of ROS, DOX could kill cancer cells at different stages of proliferation, while CA4P could inhibit the establishment of blood vessels around the tumor and increase the sensitivity of cancer cells to DOX.

### 4.6 Polymers

**FIGURE 12:**
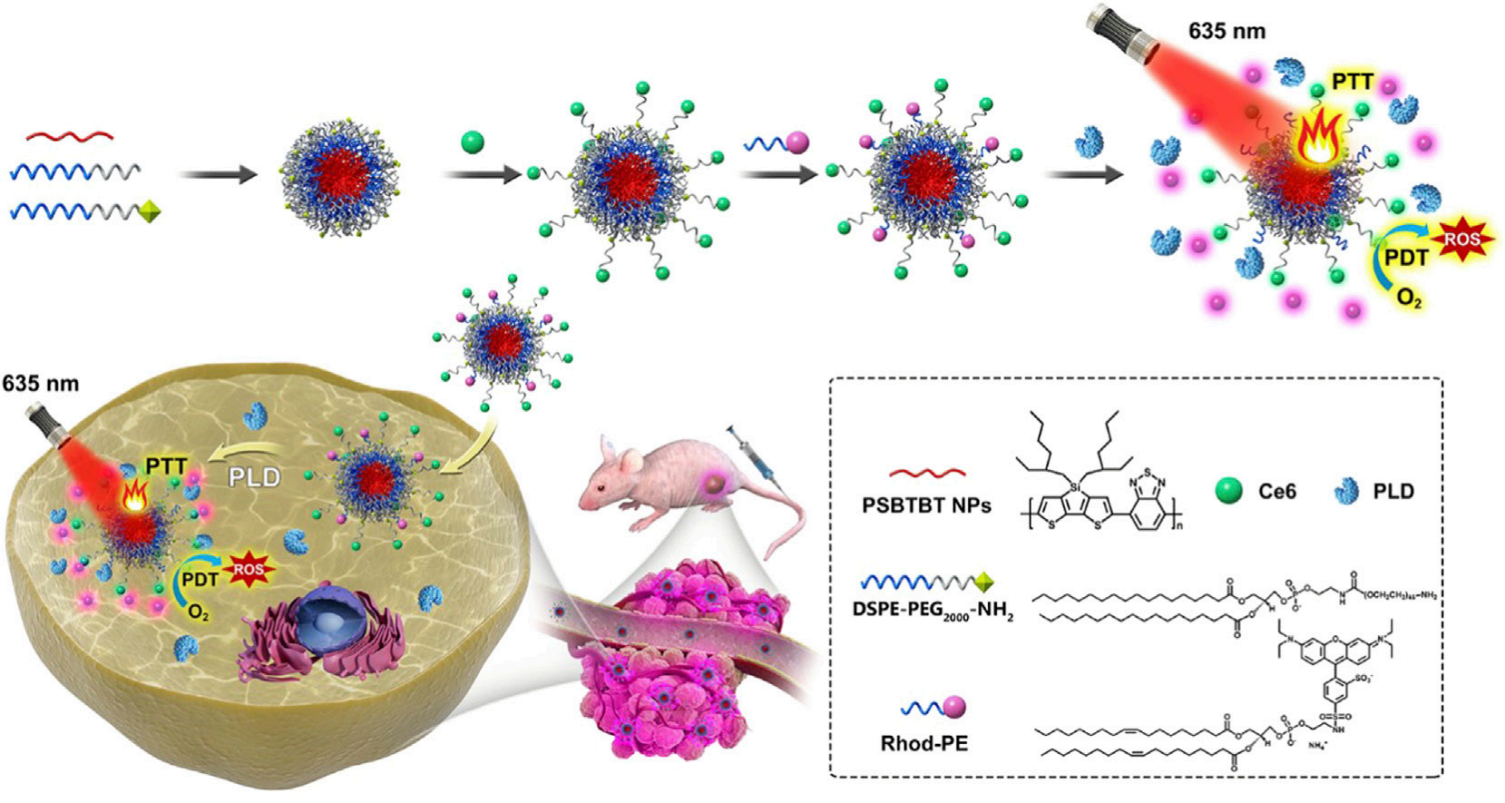
Schematic representation of PSBTBT-Ce6@Rhod NPs-mediated PLD-activatable tumor images and combined PTT/PDT therapy. PSBTBT-Ce6@Rhod NPs can kill MCF-7 cells only under light conditions. Among them, PSBTBT-Ce6 NPs mediate PTT/PDT synergistic therapy, and can also cleave Rhod in response to overexpressed phospholipase D (PLD) in tumor tissue, resulting in the fluorescence recovery of Rhod B, thereby exerting biomarker-triggered fluorescence imaging and targeting PDT. Reproduced with permission from (Bao et al., 2021).

Due to the designability and diversity of composition, structure and function, polymers have become one of the preferred carrier materials for nanomedicine. Conjugated polymers can control tissue penetration by adjusting the conjugation length. Among them, the integration of multichromophoric conjugated polymer base NPs into PSs enhanced ROS generation by improving PSs solubility, permeability, and targeting (Jiang and McNeill, 2017). According to the characteristics of carbon-based fluorescent nanomaterials such as carbon dots (CDs) and carbon-based polymer dots (CPDs) with good biocompatibility and high fluorescence yield, Sajjad et al. conjugated green-emitting CPDs to PPa to enhance the photocatalytic performance of PSs through covalent and π-π interactions Sajjad et al. (2022). Semiconducting polymer NPs (PSBTBT NPs) can’t only load PSs, but also act as photothermal agents for PDT/PTT synergistic therapy. Inspired by “biomarker-triggered image”-guided therapy, Bao et al. loaded the fluorescence quenchers Rhodamine B (Rhod B) and Ce6 on PSBTBT NPs to prepare a smart “Sensing and Healing” nanoplatform for PTT/PDT combination therapy (PSBTBT-Ce6@Rhod NPs) (Figure 12) Bao et al. (2021). Similarly, IR780 iodide can generate heat and oxygen for PDT/PTT synergistic therapy. In addition, it has strong fluorescence intensity and inherent specificity for various tumor cells, which is suitable for near-infrared imaging of tumor cells. In view of this, Potara et al. used temperature-sensitive block copolymer Pluronic F127 to wrap IR780 iodide, and coupled folic acid (FA) to Plu-IR780 micelles through chitosan to designe a thermoresponsive and thermal reversible size distribution and spectral properties of a novel FA-targeted near-infrared phototherapy NPs (Plu-IR780-chit-FA) Potara et al. (2021). Plu-IR780-chit-FA not only retained the PTT, PDT and NIR imaging properties of IR780 iodide, but also adjusted the size of the nanocapsules to the smallest (30 nm) at physiological temperature to ensue cellular uptake, while also had maximum absorption and fluorescence emission intensity.

Polymeric micelles, which constructed by amphiphilic polymers, have been regarded as ideal carriers for nanomedicine due to their good biocompatibility, degradability, easy modification of the structure, and special “core-shell” structure (Tian and Mao, 2012). Using perfluorocarbons (PFCs) as oxygen carriers to directly deliver oxygen to tumors is a common way to relieve tumor hypoxia and enhance the PDT effect. Recently, Tseng et al. developed a folate-conjugated fluorinated polymeric micelle (PFFA)-Ce6 micellar system, which exhibited a higher ROS production, good long-term stability, higher oxygen carrying capacity and improved PDT efficacy in inhibiting tumor growth as compared to those of non-PFC system Tseng et al. (2021). The fluorinated segment in PFFA-Ce6 could not only maintain the oxygen carrying capacity of polymer micelle without the problem of PFC molecule leakage, but also acted as a reservoir to accommodate the hydrophobic Ce6 to enhance its solubility. The folic moiety in PFFA-Ce6 provided a function as a specific targeting ligand for cancer cells. In the *in vitro* cell study, due to the selective internalization of PFFA-Ce6, the cell growth inhibition of HeLa cells after irradiation was higher. Cancer cells are accustomed to oxidative stress caused by PDT through overexpressing glutathione (GSH) and other antioxidants. Therefore, preferentially amplifying the oxidative stress of tumor cells by consuming GSH or producing ROS is a reasonable treatment strategy to enhance the efficacy of PDT. To this end, Xia et al. designed a GSH-scavenging and ROS- generating polymeric micelle mPEG-S-S-PCL-Por (MSLP), which was composed of methoxy polyethylene glycol (MPEG)-SS-poly (ε-caprolactone)-protoporphyrin (POR) amphiphilic polymer and the anticancer drug DOX, for amplifying oxidative stress and enhanced anticancer therapy of PDT Xia et al. (2021). MSLP combined Chemotherapy-photodynamic therapy (Chemo-PDT)-based synergy therapy exhibited significant antitumor activity both *in vitro* (IC50 = 0.041 μg/ml) and much better antitumor efficacy than that of mPEG- PCL-Por (MLP) micelles *in vivo*. In addition, Yang et al. also designed a GSH-responsive dual receptor targeting nanomicelle system, which could be used for precise fluorescent bioimaging and superior synergistic chemo-phototherapy of tumors Yang Y. et al. (2021). IR780/PTX/FHSV micelles were composed of amphiphilic hyaluronic acid derivative (FHSV), paclitaxel (PTX) and photosensitizer IR780 iodide (IR780). Once they accumulated at the tumor site through enhanced permeability and retention (EPR) effects, IR780/PTX/FHSV micelles could effectively enter tumor cells through receptor-mediated endocytosis, and then rapidly release PTX and IR780 in the GSH-rich tumor microenvironment. Under near-infrared laser irradiation, IR780 generated local high temperature and sufficient reactive oxygen species to promote tumor cell apoptosis and necrosis. The results of *in vivo* and *in vitro* experiments consistently shown that compared with single chemotherapy and phototherapy, IR780/PTX/FHSV micelle-mediated chemo-phototherapy could more effectively synergize anti-tumor effects to kill tumor cells. However, further clinical applications of polymers are limited due to the disadvantages of poor storage stability, potential tissue toxicity, limited loading capacity for hydrophilic drugs, and complicated preparation processes (Deng et al., 2021).

## 5 The Applications and the Photosensitizers for Clinical Tumor Treatment

PDT has been used to treat a variety of cancers, including lung, head and neck, brain, pancreas, peritoneal cavity, breast, prostate, skin, and liver cancer (Kubrak et al., 2022).

The first-generation PS are hematoporphyrin derivative. They have long plasma half-life and lack of sensitivity. For example, Photofrin® has been licensed for use in the oesophagus, lung, stomach, cervix and bladder. Since the absorption spectrum of Porfimer sodium peaks at 405 nm, its depth of action is limited to 0.5 cm (Ramsay et al., 2021).

The second-generation PSs are single compound synthesized, derived from porphyrin, bacteriochlorophyll, phthalocyanine, chlorin, benzoporphyrin, curcumin, methylene blue derivatives, etc., Compared with the first-generation PSs, the second-generation PSs have longer absorption spectrum (visible-near-infrared region), higher ^1^O_2_ yields, and better tumor targeting. For example, 5-aminolevulinic acid (5-ALA) has been successfully used in the treatment of basal cell carcinoma, actinic keratosis and oral premalignant disorder, Meso-tetrahydroxyphenyl chlorin (mTHPC-Foscan®) has been commonly used for advanced head and neck cancers (Zhang S. et al., 2022). Other second-generation PSs that have received clinical approval or are in clinical trials include Temoporfin (Foscan®), Motexafin lutetium, Palladium bacteriopheophorbide, Purlytin®, Verteporfin (Visudyne®), and Talaporfin (Laserphyrin®) (Baskaran et al., 2018).

The third-generation PS are characterized by combining the second-generation PSs with targeting entities or moieties, such as antibodies, amino acids, polypeptides, or by encapsulation into highly biocompatible nanocarriers to improve the ability of PS improve accumulation of PSs at the targeted tumor sites (Baskaran et al., 2018). Now, third-generation PSs that can significantly improve cancer targeting efficiency through chemical modification, nano-delivery systems, or antibody conjugation are widely studied for preclinical studies, and if satisfactory results are obtained, then more third-generation PSs will be promoted to enter the clinical research stage (Mfouo-Tynga et al., 2021).

## 6 Conclusion and Perspective

In summary, we have introduced the advantages and disadvantages (Tables 1, 2)and recent studies (Table 3) based on Metal NPs, Nanoliposomes, MSNs, Dendrimers, Hydrogels, Polymers, which overcome the obstacles of PDT in tumor tissues, such as poor biocompatibility and low delivery efficiency of PSs, compromising poor light transmittance of deep tissues, and hypoxia, reactive oxygen species scavenging and immunosuppression in tumor TME (Lee et al., 2022). Insufficient supply of pivotal factors including PSs, light, and O_2_ highly reduces the therapeutic efficacy of PDT. Therefore, the primary source of photodynamic therapy should be optimized by setting parameters such as input dose, intratumoral drug level, light source, and tissue oxygen conditions. Based on recent progress in the combination of nano- and biotechnology, various kinds of NPs have been developed for PDT, and they showed promising potential to overcome these obstacles. PSs are an indispensable key ingredient in photodynamic therapy. In most cases, PSs are aggregated inside the nanoparticles, and ^1^O_2_ has the disadvantages of short half-life (<40 ns) and short intracellular distance (<20 nm), resulting in ^1^O_2_ needing time and space to diffuse out from NPs to attack cells of biomolecules and further impairs the efficiency of PDT (Deng et al., 2021). Therefore, it is very necessary to release free PSs from nanoparticles before irradiating cancer cells or tumor tissue with light. In addition to the optimization of the physical structure and chemical composition of passive targeting of PSs and the modification of active targeting ligands for efficient, safe, and better tissue penetration, improving drug delivery and combination therapy are also effective strategies. In recent years, stimuli-responsive nanomaterials have received increasing attention because they can react to both endogenous stimuli (low pH, enzymes, redox agents, hypoxia) and exogenous stimuli (light, temperature, magnetic field, ultrasonic) and other stimuli to change its physicochemical properties and release PSs (Chang et al., 2021). However, the release of PSs from endogenous stimuli-responsive nanocarriers is slow due to weak stimulation intensity. In contrast, exogenous stimuli are easily modulated remotely in intensity and timing to release drugs on demand in diseased tissues or cells. However, the poor penetration of drugs into deep tumor tissue and the poor penetration of excitation light source into deep tumor tissue are important issues that need to be solved for NPs delivery systems. The use of UCNPs, XPDT, and fluorescence imaging techniques provides clues for efficient light delivery to deep tissues. Among them, the potential toxicity of heavy metals is a serious limitation, and their potential toxicity needs to be carefully considered, and favorable distribution, degradation and excretion should be achieved (Medici et al., 2021). Attempts to overcome hypoxia caused by hypoxia in tumor tissues include artificial oxygen production, Fenton reaction, and the combined application of chemical drugs related to hypoxia. In addition to promising results, combined with the latest methods to normalize tumor blood vessels and reduce hypoxia itself, it is also expected to become an effective method for PDT to treat tumors. Although many of these ideas are at the level of cell experiments and animal experiments, these studies have made significant progress over traditional PDT research. Therefore, we hope that the results of these trials can maximize the clinical efficacy of PDT in the future. In addition, future research and development of new nanomaterials should focus on targeted therapy and personal medicine. In order to improve the specificity and safety of drugs, targeted delivery and on-demand controlled/triggerable release are still the focus of drug delivery platform development.

**TABLE 1:** Summary of the advantages and disadvantages of Metal NPs.

Metal NPs	Advantages	Disadvantages
Au based NPs	●Utilization for PTT, PAI	●Limited stability under aqueous conditions
	●Controllable size and structure and easy surface modification	
	●Optical quenching ability	
	●Chemical inertness and excellent biocompatibility	
Ag based NPs	●Tuning optoelectronic properties according to size and shape	●Ag NPs with diameters less than 200 nm are prone to aggregation
	●High ^1^O_2_ yield	
Cu based NPs	●High photothermal conversion efficiency	●Potential toxicity
	●Low price	
	●Simple synthesis	
	●Controllable morphology and size	
	●Microwaves-induced PDT	
Ru based NPs	●Low-lying excitation energy states and high ROS yield	●Dark toxicity
	●Good photophysical and photochemical properties	●DNA mutation
	●Controllable photophysical properties	●Being excited only by short-wave visible light
	●Low photobleaching rates	
	●High water solubility	
Ir based NPs	●Unique oxygen quenching pathway	●Most Ir complexes are water-insoluble
	●Excellent electrocatalytic performance	
	●Long triplet state lifetime and good photophysical properties	
	●Significant tumor targeting ability	
Metal oxide-based NPs	●Utilization for PDT, PTT	●Limited stability under aqueous conditions
	●Clinical used MRI contrast agent	●Toxicity accumulation of NPs
	●Magnetic hyperthermia and PAI	●Physical damage from magnetic guidance
	●Easy surface modification	
	●High photostability	
	●Large extinction coefficient	
	●High emission quantum yield	
UCNPs	●Utilization for PDT, PTT, bioimaging, diagnosis, and therapy	●Potential toxicity
	●Narrow emission bandwidth, large decay time, resistance to photobleaching, and no autofluorescence background	●Limited biodegradability
	●Unique optical property and utilization for luminescence imaging	●Low drug loading capacity
	●Easy surface modification and functionalization	●Low quantum yield and superheating effects under 980 nm light source
	●Ability to absorb light in the NIR region	
Carbon-Based NPs	●Strong optical absorbance and utilization for PTT, PAI	●Induce inflammatory reactions and cytotoxicity
	●Unique electrical property	●Limited biodegradability
	●Easy surface modification	●Low utilization of visible light
	●High surface-to-volume ratio	●Expensive and complex synthetic method
	●Thermal stability	
	●High photoluminescence quantum yield	
Sulfur-based NPs	●Utilization for PTT, CDT, PDT	●The degradation products have potential toxicity
	●Good biocompatibility	●Killing efficiency on hypoxic tumor cells is limited
	●High photothermal conversion efficiency	
	●Cheap and simple manufacturing method	
	●Biodegradability and rapid metabolism	
Phosphorus-based NPs	●Optical and electrical properties better than carbon-based metal NPSs and sulfur-based metal NPSs	●Weak absorption in the biowindow and low photo catalytic activity in a TME
	●For making photosensitizers	●The inherent instability of BP NSs and BP QDs in water–air environments
MOFs	●Facile diffusion of ROSs through their porous structures	●Complex design, lengthy preparation steps and high operating costs
	●High specific surface area	●Early clearance by body immune system
	●Controllable size, shape and function of the pore	●Off-target accumulation
	●Effectively enhance the ROS generation effect	●Untimely drug release ability
	●High PSs loadings	

**TABLE 2:** Advantages and disadvantages of other representative nanomaterials.

Type	Advantages	Disadvantages
Nanoliposomes	●Biocompatibility and biodegradability	●Low drug loading capacity
	●High structural flexibility	●Limited stability *in vivo*
	●Targeted delivery and triggered release	●Uncontrolled drug leakage
	●Easy and diverse surface modification	●The inevitable self-quenching effect of water-insoluble PSs
	●Prolonged tissue penetration and retention of PSs	
MSNs	●Large specific surface area and huge specific pore volume	●The larger the specific surface area, the greater the cytotoxicity
	●Easy and diverse internal and external surface modification	
	●High loading efficiency	
	●High targeting	
Dendrimers	●Controllable molecular size	●High molecular weight, high-density surface positive charge increases the toxicity of dendrimers
	●Large number of terminal functional groups	
	●Large number of cavities in the molecule	
Hydrogels	●Good biocompatibility	●Low mechanical strength
	●Efficient adhesion to biotic surfaces	●Poor repeatability of material properties
	●Delivery of hydrophilic drugs	
Polymers	●The designability and diversity of composition, structure and function	●Limited storage stability
	●Diverse surface modification	●Potential toxicity
	●High loading efficiency and sustained release	●Limited loading capacity for hydrophilic drugs
	●Good circulation stability	●Complex synthesis process
	●Improve PSs solubility, permeability, and targeting	

**TABLE 3:** Summary of recently developed NPs to overcome the obstacles of current photodynamic therapy in tumor.

Obstacles to overcome	NP type	Name	Strategy	Year
PSs delivery	DNA-modified NPs	Apt-DNA-Au nanomachines (Yu et al., 2021)	Tumor-associated TK1 mRNA-responsive PSs release and survivin targeting by antisense DNA	2021
	DNA-modified NPs	TCPP-gDNA-Au/PLNP (Su et al., 2021)	Nucleolin targeting by AS1411 aptamer	2021
	DNA-modified NPs	Au/Pd ONP-DNA nanomachine (Cai et al., 2021a)	Using the primary marker miRNA-21 and two auxiliary markers miRNA-224 and TK-1 mRNA to improve the accuracy of tumor identification	2021
	DNA-modified NPs	Label-rcDNA-AuG (Zhang et al., 2021b)	Recognition of cancer cells by miR-21	2021
	Biotin-modified NPs	BT@Au-NPs (He et al., 2021b)	Movement to cellular sites and efficient binding sites in tumor cell lines by biotin	2021
	AuNRs-grafted RGD	HB-AuNRs@cRGD (Liu et al., 2021f)	Binding of RGD to integrin avb3 in tumor cells and tumor neovascular endothelial cells	2021
	Au nanoshells	40/20 core radius/shell thickness optimized gold nanoshell (Farooq and de Araujo, 2021)	Optimization of nanoshells structure (silica core radius and gold shell thickness) to increase the singlet oxygen production	2021
	Heterometallic colloids	(L' = I−, CH_3_COO−) Mo_6_Au_2_ colloids (Faizullin et al., 2021)	Affecting NPs cytotoxicity, cellular internalization, and PDT activity by modulating the order of supramolecular stacking by Mo_6_-Au_2_	2021
	Polymer-coated AuNRs	Au-MB-PEG NPs (Liu et al., 2021g)	Response to highly expressed HOCl in the tumor region *via* FDOCl-24	2021
	Cu-based Fenton reagents	Cu-LDH/HMME@Lips (Wu et al., 2021)	Active infiltration of cancer cells by Cu-LDH for deep tumor therapy. Extended circulatory residence time by liposome encapsulation	2021
	Hollow mesoporous silica supported UCNPs	UCNP/RB@mSiO2-NH (Chen et al., 2021a)	“One treatment, multiple irradiation” PDT strategy for efficient nuclear-targeted PDT	2021
	Metal-organic frameworks	Zn (II)-PPIX/G-quadruplex VEGF aptamer-tetrahedra structures (Zhang et al., 2021c)	Release of PSs in response to VEGF *via* VEGF-aptamer-functionalized DNA tetrahedra	2021
	Nanoliposomes	Fru-Bio-Lip (Li et al., 2021b)	Increased total number of liposomes bound to cancer cells by dual-ligand modification of fructose and Bt	2021
	Fluorinated dendrimer	APFHG (Zhu et al., 2021a)	EGFR-TKI specifically recognizes EGFR-positive NSCLC cells and releases Gef and Hp in response to a hypoxic acidic microenvironment	2021
	Polyamidoamine Dendrimers	G5MEK7C(n)-ICG (Cui et al., 2021)	p (EK) converts to positive charge in response to acidic TME and interacts more readily with tumor cell membranes	2021
Light delivery	Bimetallic NPs	Au-BiGSH@IR808 (Jia et al., 2021)	Modified by IR808 fuel for higher NIR photon capture capability	2021
	Ultra-thin two-dimensional nanosheets	4-layer O-Ti_7_O_13_ nanosheets (Dai et al., 2021a)	X-ray irradiation-induced ROS generation by OTi_7_O_13_ nanosheets and chemotherapy mediated by DOX	2021
	Ti-based targeting agent	B-TiO_2_@SiO_2_-HA (Guo et al., 2021)	Simultaneous generation of ROS and hyperthermia under NIR-II laser irradiation and full spectral response to light stimulation obtained by B-TiO_2_	2021
	Semiconductor metal oxide	SnO_2-x_@SiO_2_-HA (Gao et al., 2021a)	SnO_2-x_-mediated full-spectrum response target-specific synergistic PDT/PTT	2021
	Block copolymer	Plu-IR780-chit-FA (Potara et al., 2021)	PTT/PDT synergistic therapy under NIR *via* IR780	2021
	UCNP	UCNP/RB, Ce6 (Pham et al., 2021)	Dual PSs have higher PDT efficiency than single PS	2021
New PSs	Ru complex	Ru-I (He et al., 2021c)	Red-Light-Responsive Ru Complex PSs for lysosome localization PDT	2021
	Amphiphilic polymer	DSPE-PEG_2000_-Folic encapsulated Ru (II) polypyridine complex (Karges et al., 2021)	Enhanced tumor cell selectivity by DSPE-PEG_2000_-Folic	2021
	Ru (II) complex	Ru (II) complex-based bioorthogonal two-photon PSs (Lin et al., 2021a)	Anti-tumor effects by specifically binding to cancer cell membranes and inducing cell membrane damage	2021
	Ir compounds	Ir (III) complexes (Xiao et al., 2021)	Different degrees of oxygen quenching *via* Ir (III) complexes	2021
	Bifunctional Ir (III) complexes	4 ([Ir(Bzq)_2_ (dpa-acr)]+ (Redrado et al., 2021)	Targeted mitochondrial and cellular imaging *via* organic chromophores and Ir (III) complexes	2021
	Superparamagnetic Fe_3_O_4_ NPs	E-NP (Sengupta et al., 2022)	E-NP show immunoprotective and anti-inflammatory effects by inhibiting MPO and down-regulating NO	2022
	Ru (II) polypyridine complexes	Ru-g-C_3_N_4_ (Sengupta et al., 2022)	Oxygen self-sufficient PSs generated by grafting metal complexes onto g-C_3_N_4_	2021
	Graphitic carbon nitride	g-C_3_N_5_NSs (Liu et al., 2021h)	Due to the addition of nitrogen-rich triazole groups, the visible light utilization and photocatalytic activity of g-C_3_N_5_NSs are higher than those of g-C_3_N_4_NSs	2021
	nanoheterostructures	Ni_3_S_2_/Cu_1.8_S@HA (Sang et al., 2021)	Production of ROS and O2 by Ni_3_S_2_/Cu_1.8_S	2021
	BPQDs	BPQDs@PEI + RGD-PEG + DMMA (Liu et al., 2021i)	Enrichment of tumor targets through pH-responsive charge switching	2021
	2D black phosphorus nanosheets	Cyan@BPNSs (Qi et al., 2021)	Continuous oxygen supply through cyanobacterial photosynthesis	2021
	Red/black phosphorus composite nanosheet	M-RP/BP@ZnFe_2_O_4_ (Kang et al., 2022)	ZnFe_2_O_4_ enhances the productivity of ROS through the Fenton reaction and can also induce apoptosis in MB-231 cells through oxidative stress	2022
	Carbon-based polymer dots	PPa-CPD (Sajjad et al., 2022)	PPa enhances the photocatalytic performance of photosensitizers *via* covalent and π-π interactions	2021
Unfavorable TME	Hyaluronic acid-Bimetallic NPs	ToHAu@Pt-PEG-Ce6/HA (Bu et al., 2021)	Oxygen enrichment in tumor and PDT by Pt	2021
	Bimetallic NPs	Au/Ag NR (Jin et al., 2021b)	Increases heat and ROS production by altering the amount of Ag^+^, triggering ICD in tumor cells	2021
	lateral nano-heterostructure	(Bi/BiO_x_)-based lateral nano-heterostructure (Qiu et al., 2021)	Oxygen-independent PDT using BiO_x_	2021
	Nanozyme	IrO_2_-Gox@HA NPs (Yuan et al., 2022a)	Enhancement of type II PDT by GOx and IrO_2_ NPs	2022
	ZGGO durable luminescent NPs	Mn-ZGGO (Ding et al., 2021b)	Oxygen-independent PDT using MnO_x_ shell	2021
	Nanozyme	ICG@PEI-PBA-HA/CeO_2_ (Zeng et al., 2021)	CeO_2_ catalyzes H_2_O_2_ to O_2_ through Ce^3+^/Ce^4+^ cerium valence cycling	2021
	UCNPs	UCTM NPs (Cheng et al., 2021a)	Oxygen-enriching role of thylakoid membranes of chloroplasts in tumors and photodynamic therapy	2021
	UCNPs	CM@UCNP-Rb/PTD (Jin et al., 2021a)	PEG-TK-DOX releases DOX in response to ROS and prevention of tumor metastasis by CD73 antibody	2021
	MIPs modify UCNPs	MC540/MNPs@MIPs/UCNP (Lin et al., 2021b)	Using MIPs to target tumor cells and prevent PD-1/PD-L1 immune blockade	2021
	Molybdenum Carbide	Mo_2_C@N-Carbon-3@PEG (Hou et al., 2022)	Photocatalytic Oxygen Generation by Mo_2_C	2022
	Engineered bacteria	EB (Ding et al., 2021a)	Targeting anoxic TME and catalyzing H_2_O_2_ to produce O_2_ using engineered *Escherichia coli*	2021
	Metal-organic frameworks	UIO@Ca-Pt (Ren et al., 2021)	Increase intracellular oxygen content by endogenous oxygen through CaO_2_ and Pt	2021
	Nanoscale iron-based metal organic frameworks	MIL-101(Fe)@TCPP (Chen et al., 2021b)	Fenton reaction increases intracellular oxygen levels	2021
	Metal Organic Framework Nanosystems	NMOF@SF/TPZ (NST) (Yu et al., 2022)	Disturbed redox metabolism in tumor cells caused by GSH depletion and Fenton reaction oxygen enrichment	2021
	Metal-organic frameworks	Ag-AgCl@Au NMs (Liu et al., 2021j)	Au nanorods produces O_2_ through a photocatalytic reaction	2021
	Nanoliposomes	Ce6-SB3CT@Liposome (Lip-SC) (Liu et al., 2021a)	The released SB-3CT can effectively activate NK cells and enhance the immune system by inhibiting the shedding of soluble NKG2D ligands	2021
	Double nanozyme modified HMSN	HMSN@Au@MnO_2_-Fluorescein Derivative (HAMF) (Chen et al., 2021c)	Enhancement of intracellular oxygen level by catalytic reaction of MnO_2_ and Au NPs	2021
	Double nanozyme modified HMSN	AuNCs@mSiO_2_@MnO_2_ (Yin et al., 2021)	Acid-TME-responsive dual nanozyme-catalyzed reaction to enhance intracellular oxygen level *via* MnO_2_ nanosheets	2021
	Polyamidoamine Dendrimers	CCT-DPRS (Mindt et al., 2018)	CaF_2_NPs convert low-dose X-radiation to Wei-green light to excite Rb to generate ROS, while releasing SU to inhibit tumor angiogenesis	2021
	Hydrogels	OPeH (Liu et al., 2021b)	MnO_2_ NPs convert H_2_O_2_ to O_2_, which further promotes the generation of ^1^O_2_ from PpIX and improves the generation efficiency of ^1^O_2_	2021
	Fluorinated polymer micelles	(PFFA)-Ce6 (Tseng et al., 2021)	Using perfluorocarbons to increase intracellular oxygen levels	2021
	Amphiphilic polymer micelles	MPEG-S-S-PCL-Por (MSLP) (Xia et al., 2021)	Amplifies oxidative stress in tumor cells by depleting GSH and producing ROS	2021
Synergistic therapy	layered double hydroxides	ICG/CAC-LDH (Wang et al., 2021a)	Induces intracellular GSH depletion through redox reactions, and can also be decomposed to generate Cu+ and Ce3+, which stimulates Fenton-like reactions to generate OH	2021
	Bimetallic NPs	Au_1_Bi_1_-SR NPs (He et al., 2021a)	The photothermal effect of NPs is enhanced by the introduction of Bi	2021
	Bifunctional micelles	Micelle-Ir (Liu et al., 2021c)	Promotion of singlet oxygen generation and photothermal effect via BODIPY-Ir	2021
	Nanozyme	MIP/Ce6 (Li et al., 2021a)	PTT by IrO_2_ and TME-responsive PDT by MnO_2_	2021
	zeolitic imidazole framework-67 NPs	Co_3_S_4_-ICG (Jiang et al., 2021b)	Promoting Fenton reaction to generate ROS through PTT	2021
	Bovine serum albumin (BSA) NP	FeS_2_@SRF@BSA (Feng et al., 2021)	The combination of Fenton-like reaction and PDT enhanced ROS production and antitumor effect	2021
	Metal-Organic Framework	Zr-MOF@PPa/AF@PEG (Wang et al., 2021b)	Zr-MOF@PPA/AF@PEG take advantage of the PDT-induced hypoxia to activate HIF-1 inhibitor AF to enhance the anti-tumor effect and achieve the synergistic PDT- chemotherapy (PDT-CT) therapeutic effects	2021
	Metal-Organic Framework Core-Shell Hybrid Materials	Au@MOF-FA (Cai et al., 2021b)	Fe_3_O(OAc)_6_(H2O)^3+^-mediated Fenton reaction and Au nanorod-mediated PTT	2021
	Nanoliposomes	Lip(PTQ/GA/AIPH) (Dai et al., 2021b)	PTDT/PTT/PDT synergistic therapy *via* PTT, PTDT prodrug and GA	2021
	PEGylated MSNs	M(A)D@PI-PEG-RGD (Zhang et al., 2021d)	Synergistic treatment of chemotherapy, PTT and PDT by ICG and DOX	2021
	Phenylboronic acid modified dendrimers	P-NPs (Zhong et al., 2021a)	Synergistic chemophotodynamic therapy that releases PTX in response to high concentrations of glutathione and H_2_O_2_ in tumor cells increases intranuclear PSs through nuclear membrane disassembly	2021
	Hydrogels	DOX-CA4P@Gel (Zhong et al., 2021b)	The gel can be slowly degraded under acidic TME, and DOX and CA4P are released in different time sequences for tumor therapy	2021
	Polymer micelles	IR780/PTX/FHSV micelles (Yang et al., 2021a)	Release of PTX and IR780 in response to GSH for chemophototherapy	2021
“ Sensing and Healing” nanoplatform	Bimetallic NPs	Au-AgNP-Ag-HM (Wang et al., 2021c)	The imaging of intracellular caspase-3 and ROS by DEVD and Au-Ag-HM differentiates cancer cells from normal cells	2021
	Semiconducting polymer NPs	PSBTBT-Ce6@Rhod NPs (Bao et al., 2021)	PSBTBT NPs loaded with Rhodamine B and Ce6 for combined PTT/PDT therapy	2021
